# Causal effects of education on sexual and reproductive health in low and middle-income countries: A systematic review and meta-analysis

**DOI:** 10.1016/j.ssmph.2019.100386

**Published:** 2019-05-20

**Authors:** Stephanie R. Psaki, Erica K. Chuang, Andrea J. Melnikas, David B. Wilson, Barbara S. Mensch

**Affiliations:** a Poverty, Gender and Youth Program, Population Council, New York, NY, USA; b Criminology, Law and Society, George Mason University, Fairfax, VA, USA

**Keywords:** Education, Sexual and reproductive health, Systematic review, Meta-analysis, Low and middle-income countries

## Abstract

**Background:**

Despite strong theoretical grounding, important gaps in knowledge remain regarding the degree to which there is a causal relationship between education and sexual and reproductive health, as many claims have been made based on associations alone. Understanding the extent to which these relationships are causal is important both to inform investments in education and health, as well as to understand the mechanisms underlying these relationships.

**Methods:**

We conducted a systematic review of the evidence for a causal link between education and sexual and reproductive health (SRH) in low and middle-income countries. Education indicators included exposure to formal schooling and learning. SRH outcomes included: age at first sex, age at first marriage, age at first pregnancy/birth, contraceptive use, fertility, and HIV status and other sexually transmitted infections. When possible, we also conducted meta-analyses to estimate mean effects by outcome, and to understand sources of variation between studies.

**Results:**

We identified 35 papers that met our inclusion criteria. Although many of the studies report evidence of a causal relationship between education and one or more SRH outcomes, estimated effects are often small in magnitude. Our meta-analyses reveal mostly null mean effects, with the exception of small effects of increased grade attainment on lower fertility and HIV positive status. We also found inconsistent evidence supporting mechanisms linking education and SRH.

**Conclusions:**

This review demonstrates that, although investments in schooling may have positive ripple effects for sexual and reproductive health in some circumstances, those effects may not be as large or consistent as expected. Further, our understanding of the circumstances in which schooling is most likely to improve SRH remains somewhat limited. An accurate picture of whether and when improvements in education lead to better health outcomes is essential for the achievement of global development goals.

## Highlights

• Many authors reported small effects of education on sexual and reproductive health.
• Most mean effects of education on sexual and reproductive health were null.
• We find small effects of increased grade attainment on fertility and HIV status.
• Evidence for mechanisms linking education and sexual and reproductive health is inconsistent.

## 1 Background

In the last several decades, low and middle-income countries have experienced rapid expansions in access to primary school. Alongside strong policy commitments and financial investments (Hewett & Lloyd, 2005; Psaki, McCarthy, & Mensch, 2017), between 1970 and 2010 primary school enrollment increased from 68 percent in sub-Saharan Africa and just under 50 percent in South Asia to near universal levels in both regions (World Bank, 2018). At the same time, many countries have seen improvements in sexual and reproductive health indicators, including declines in fertility and child marriage (Lloyd, Kaufman, & Paul, 2000; Nguyen & Wodon, 2012). Researchers point to the parallel trends of increasing access to formal schooling and improving sexual and reproductive health as evidence of links between the two (Cleland, 2010; Gakidou, Cowling, Lozano, Christopher, & Murray, 2010; Lloyd et al., 2000), and continued investments in global education are often justified, in part, by expectations of positive ripple effects for health.

A vast literature exists exploring the linkage between formal education, particularly for females, and health outcomes in low and middle-income countries (LMICs). Numerous studies have documented and analyzed the inverse association between years of schooling attained, particularly by women, and early sexual initiation and marriage, as well as levels of fertility and maternal, infant and child mortality (Basu, 2002; Cleland & van Ginneken, 1988; Glewwe, 1999; LeVine, LeVineSchnell-Anzola, Rowe, & Dexter, 2012). Literature documenting the inverse association between educational attainment and HIV infection risk also argues that education acts as a “social vaccine” to prevent the spread of infection (Behrman, 2015a, Behrman, 2015b; De Neve et al., 2015a, De Neve et al., 2015b). However, despite enormous progress in expanding access to formal schooling in recent decades, improvements in key sexual and reproductive health indicators have not always been commensurate (Bongaarts & Casterline, 2013; Mensch, Singh, & Casterline, 2005).

A substantial theoretical literature lays out the possible pathways linking education and sexual and reproductive health.^1^ These theories highlight two main pathways: education's effects on changing preferences (e.g. fertility, timing of marriage), and on changing women's and men's abilities or opportunities to achieve their preferences (e.g. income, knowledge and use of contraception including condoms). Some have argued that education alters household fertility preferences by increasing the cost of children and transforming norms around family size and the role of children (Bledsoe and Cohen, 1993; Caldwell, 1980). Others have postulated that education increases autonomy, especially for women, enabling them to achieve their preferences by delaying marriage or negotiating family planning decisions with their partners, and empowering them to engage effectively with healthcare providers (Bledsoe, Casterline, & Johnson, 1999; Jejeebhoy, 1995; LeVine et al., 2012). Education may expand opportunities to engage in the labor market, thereby changing preferences through increased opportunity cost of childbearing for women (Jejeebhoy, 1995; Lloyd & Mensch, 1999). Education may also change parents' abilities to provide for their children financially, and to engage with the healthcare system when their children are sick (LeVine et al., 2012) leading to lower infant and child mortality, and subsequently lower desired fertility (Gakidou et al., 2010; Shapiro and Tenikue, 2017). For adolescents, education may have an “incarceration” effect, leading to delayed marriage and childbearing while enrolled in school (Black, Devereux, & Salvanes, 2008). More educated women may engage in “assortative mating”, finding partners who are also more educated, and may have similar fertility preferences (Basu, 2002).

Despite strong theoretical grounding, important gaps in knowledge remain regarding the degree to which there is a causal relationship between education and sexual and reproductive health, as many claims have been made based on associations rather than causal relationships. At the population level, improvements in education might coincide with improvements in sexual and reproductive health due to factors such as economic development leading to investments in school and healthcare infrastructure, political stability following periods of conflict or uncertainty, or changes in attitudes and norms due to increased exposure to mass media. At the individual level, factors such as household wealth, gender norms, or genetic endowments may contribute to both higher levels of education and better sexual and reproductive health outcomes. That is, even in the absence of a causal relationship between education and sexual and reproductive health, we might expect coinciding patterns of improvement.

Understanding the extent to which these relationships are causal, rather than attributable to a common set of underlying factors, is important both to inform investments in education and health, as well as to understand the mechanisms underlying these relationships. For example, if increased grade attainment leads to lower fertility, in what contexts is this relationship most likely to hold, and are there important thresholds for grade attainment, such as primary school completion? If literacy acquisition is necessary for improved sexual and reproductive health, what are the consequences of poor-quality schooling?

In seeking to understand the effects of education on sexual and reproductive health, we are concerned about two main sources of bias: 1) reverse causality, which can occur when estimating the relationship between school attendance and sexual debut, child marriage or adolescent childbearing; and 2) factors, many of which are often unmeasured, that affect both education and sexual and reproductive health, such as family background, tendency to plan for the future, motivation or genetic endowment. Analyses that fail to address these concerns may lead to biased estimates of the relationship between sexual and reproductive health.

To document and fill this gap in knowledge, we conducted a systematic review of the evidence for a causal link between education and sexual and reproductive health in low and middle-income countries. Education indicators included several measures of exposure to formal schooling and learning (including literacy and numeracy). Sexual and reproductive health outcomes included: age at first sex, age at first marriage, age at first pregnancy or birth, contraceptive use, fertility, and HIV status and other sexually transmitted infections (STIs). This paper is part of a larger effort to review the causal evidence on links between education and health. Results on the effects of education on maternal and child health are reported elsewhere (Mensch, Chuang, Melnikas, & Psaki, 2018).

## 2 Methods

As this systematic review was designed and implemented as part of a larger effort to assess the causal relationships between education and a series of health outcomes, we report methods for the full review when relevant. The study protocol is published online (PROSPERO registry # CRD42017073224).

### 2.1 Identification of studies

We searched selected peer-reviewed and gray literature databases for English language articles from 1990 or later using the terms included in the protocol. Search terms were designed to address topic areas of interest as well as study design and included education (e.g., educational attainment, enrollment, school dropout, learning, literacy, numeracy), and our outcomes of interest (e.g., sexually transmitted infections, HIV, age at first sex, sexual debut, age at first marriage). Study design search terms included items such as randomization, causality, experiment, instrumental variable, and regression discontinuity in an attempt to identify experimental and quasi-experimental studies.^2^ In addition to database searches, we reviewed reference lists for pertinent articles, and reviewed recommended articles from a study advisory group, as well as from authors of studies identified through our initial search.

### 2.2 Inclusion criteria

After the initial search, we removed duplicates and uploaded titles and abstracts to Covidence, an online systematic review tool, to manage the abstract and full text review process. We first reviewed article titles and abstracts to assess relevance. Two reviewers independently assessed each abstract and disagreements were discussed before referring to a third reviewer as needed. Articles were included for full text review if they met the following inclusion criteria, or if we could not determine whether they met one or more of the inclusion criteria (e.g. country of study was not reported):

• Any of the exposure and outcome measures were included, as noted in our protocol.
• Same exposure(s) and outcome measure(s) were analyzed for both treatment/quasi-treatment and control/quasi-control groups.
• Analysis attempted to control for endogeneity in the relationship between education and at least one health outcome(s) of interest, listed below.
• Study methods eligible for inclusion were: randomized controlled trials (RCTs), both longitudinal and repeated cross-sectional survey methods OR natural experiments that addressed the endogeneity of schooling and health or other designs that addressed the endogeneity of policy changes or programs that improve schooling and health.^3^
• Reports on data from low- and middle-income countries as defined by the World Bank.
• Published in 1990 or later.
• Published in English.

For an RCT or natural experiment to be included in our analysis, the authors had to first show that the education intervention/exposure was significantly associated with an education outcome, without which they would be unable to address the endogeneity between education and health.

### 2.3 Exclusion criteria

From the initial abstract and title screening, articles were advanced to full text review. Two reviewers independently reviewed each full text article to ensure all inclusion criteria were met. Articles were excluded according to the following criteria:

• None of the exposure and outcome measures were included, as noted in our protocol.
• Different exposure(s) and outcome measure(s) were analyzed between treatment/quasi-treatment and control/quasi-control.
• Data were exclusively qualitative.
• Quasi-experimental studies lacked a controlled comparison and/or studies did not attempt to control for endogeneity between stated education exposure(s) and health outcome(s), including those that adjusted for covariates in regression models, or used location-level fixed effects, as their only means of controlling for endogeneity of the previously stated relationship.
• For studies that employed matching, no formal matching method was stated.
• Data were from high income countries as defined by the World Bank.
• Gray literature, with the exception of working papers and book chapters.
• Studies published prior to 1990.
• Non-English language articles.

In the case of disagreements, reviewers met to discuss the article. When an agreement could not be reached, a third team member reviewed the article.

### 2.4 Data extraction

After review of the full text articles, we extracted relevant data from each included study to a shared spreadsheet. The data extraction form was designed in consultation with the Cochrane Handbook (Higgins & Green, 2011) and included study design; methods; participants; sample size; analysis methods; outcome measure(s); exposure measure(s); effect sizes; statistical significance; and discussion of mechanisms. In the course of data extraction, if additional information was needed, authors were contacted by emailing the first author at the address provided in the publication, with up to three contacts over the course of a month.

After data extraction, two reviewers (Authors 2 and 3) independently assessed study quality for each of the included articles, with a third reviewer (Authors 1 or 4) brought in to settle disagreements. Using a study quality assessment tool adapted from Baird, Ferreira, Özler, and Woolcock (2013) and GRADE, we assessed the quality of both experimental and quasi-experimental studies. Studies were assigned points based on whether they addressed six domains: 1) selection bias (analyses properly addressed sources of endogeneity); 2) methods-specific criteria (appropriate use and reporting of study design and analytical approaches); 3) sample size (calculation reported or justification given for small sample or sufficiently large sample used for secondary analyses, and statistical analysis takes sampling design into account); 4) confounding factors (based on observed variables, study controlled for potential confounding and balance was assessed between comparison groups); 5) respondent and data attrition (attrition balanced across groups and below acceptable thresholds); and 6) theory of change (authors describe any theoretical pathways linking education and health to motivate analyses). Each study was given 0 or 1 on each of the six criteria outlined, for a possible score ranging from 0 to 6. We categorized studies that scored a 4 or higher as “high quality”, those that scored a 3 as “medium quality”, and all others as “low quality” studies. Note that, given the stringent inclusion criteria for this review, we consider all papers that met those criteria to be higher quality than much of the existing literature. The distinctions within that group with regard to score assignments are largely related to the information reported by the authors, rather than fundamental flaws in study design. The full study quality assessment tool is included in our protocol, and details on how each study was rated are available in Table S1.^4^

### 2.5 Data analyses

Our analyses proceeded in several steps. First, we developed a descriptive summary of studies that met our criteria, such as information on geographic representation, study design, sample, date of publication, source of publication, and outcomes assessed. We grouped results from statistical models by the approach to measuring the outcome, which varied even between papers seeking to estimate the same relationship. Some papers included multiple models measuring the same outcome in different ways; in those cases we include the models that were most comparable to other studies. We categorized our results by outcome at the model level and identified those in which the authors reported a statistically significant result in the expected direction (that is, education improves health), a null result, or a statistically significant result in the unexpected direction (education leads to poorer health).^5^

To facilitate comparison of results across studies, we converted effect sizes from each result into partial correlation coefficients along with 95% confidence intervals. Bivariate effect sizes, including bivariate correlations (e.g. Pearson correlation coefficients), represent the association between two variables. Partial effect sizes, including partial correlations, represent the relationship between two variables (in this case, education and health), controlling for one or more additional covariates (Aloe, 2014; Aloe & Thompson, 2013). As with bivariate correlations, partial correlations are scale-free, and range in value from −1 to 1, with stronger relationships represented by estimates closer to the absolute value of 1. Guidelines for interpretation of magnitudes of correlations vary between disciplines and depend on the nature of the relationship studied. According to Cohen’s (1988) conventions for interpreting effect sizes, correlations less than or equal to 0.10 are considered small, values of 0.25 are considered medium, and values greater than or equal to 0.40 are considered large (Lipsey & Wilson, 2001). In the case of this study, which reports partial correlation coefficients that are adjusted for a set of key covariates, we might expect the magnitude to be somewhat smaller than the guidelines for bivariate correlations.

Given the diversity of variable definitions and analytical approaches in the papers identified in our review, conversion of reported effect sizes into partial correlations required numerous different approaches. Specifically, the 35 papers identified in our review included 22 different statistical model types (e.g. linear models with a continuous independent variable and a continuous dependent variable, logit models that report odds ratios), each of which called for a different approach to converting partial effects into partial correlations. Some approaches to this conversion required data that were not reported in the papers. In those cases, we contacted authors for additional information.^6^ It should be noted that for probit models, no consensus exists in the literature on conversion of regression parameters to partial correlations, so we have used rough estimates (Lipsey & Wilson, 2001). Appendix S2 includes a full list of model types and formulas used for conversion, as well as details about how each equation was applied. Outcomes that were analyzed using linear models and a continuous dependent variable were converted into partial correlations using Equation 1.1 in order to improve comparability across studies. This equation tends to dampen overall effect sizes, producing more conservative estimates of the relationships of interest. For models outside those for which we could apply Equation 1.1, such as logit and probit models, Equations 2.1–2.3 were applied. For all models we show whether the reported effect size was statistically significantly different from zero, assuming an alpha of 0.05.

We display partial correlations from models that measured the same outcome using the same approach in forest plots to visually examine the distribution of estimated effect sizes. We note cases where: 1) authors did not report the necessary data to convert estimated effect sizes to partial correlations, and we have not received that information through direct communication; or 2) methods do not exist to convert regression parameters to partial correlations. In both cases, these model results are excluded from forest plots. In cases where authors reported comparable results from both OLS models and more rigorous models designed to address endogeneity (e.g. those using instrumental variables estimation, regression discontinuity, etc.),^7^ we convert reported effect sizes from both models into partial correlations, and display both in tables and figures to show the differences between these estimates.

After converting each study's estimated effects to partial correlation coefficients, we conducted meta-regressions to estimate mean effect sizes and 95% confidence intervals for selected study outcomes. We ran random effects models based on the assumption that identified studies are likely drawn from a larger population of studies that do not have a common effect size (Lipsey & Wilson, 2001). We estimated a restricted maximum likelihood (REML) random effects model rather than the more-often employed DerSimonian-Laird (DL) model due to software limitations, but multiple studies have shown that DL and REML produce relatively comparable estimates of between-study variance (Chung, Rabe-Hesketh, & Choi, 2013; Novianti, Roes, & van der Tweel, 2014; Viechtbauer, 2005). We include meta-regressions when three or more studies were identified that: 1) drew from different study populations; 2) measured an exposure and outcome the same way; and 3) used the same study design (i.e. quasi-experimental or RCT). Mean effect sizes were calculated by weighting each study's partial correlation coefficient using the following equation:

$$w_{i,RE}=\frac{1}{\left(v_{i}+{\hat{\tau }}_{REML}^{2}\right)}$$

where $w_{i}$ is the weight of study i, $v_{i}$ represents within-study variance, and ${\hat{\tau }}_{REML}^{2}$ is the between-study variance for the REML model, as calculated below through an iterative process where the initial estimate of ${\hat{\tau }}_{REML}^{2}\geq 0$.

$${\hat{\tau }}_{REML}^{2}=max\left\{0,\frac{\sum^{}w_{i,RE}^{2}\left({\left(y_{i}-{\hat{\mu }}_{RE}\left({\hat{\tau }}_{ML}^{2}\right)\right)}^{2}-v_{i}\right)}{\sum^{}w_{i,RE}^{2}}+\frac{1}{\sum^{}w_{i,RE}}\right\}$$

Here, $y_{i}$ is the treatment effect for study i, ${\hat{\tau }}_{ML}^{2}$ is the between-study variance from the maximum likelihood model, and ${\hat{\mu }}_{RE}$ is the mean effect size under a random-effects estimation (Veroniki et al., 2016).

For each outcome where a meta-regression was possible, we report Cochran's *Q* statistic in order to assess the extent to which the included effect sizes all estimate the same population effect size (that is, from a homogenous population). Since the variation in effect sizes between studies may reflect both true heterogeneity and random error, a statistically significant *Q* indicates that the variation in study effect sizes is not due to error alone, and therefore studies are drawn from a heterogeneous population. However, a nonsignificant p-value for Q does not necessarily rule out the possibility of variations in true effects, as this may be due to low power (Lipsey & Wilson, 2001). We also report the between-study variance estimator τ,^2^ as calculated above from the REML model. In addition, we include the *I*^2^ statistic, a study-level measure of effect, which quantifies the percentage of the variance in a set of studies that is due to the studies themselves rather than sampling error. Cochrane collaboration guidelines indicate that an *I*^2^ greater than 75% suggests considerable heterogeneity between studies, while a value below 40% suggests that heterogeneity may not be important (Cooper, 2017).

Based on the results from the homogeneity analyses, as a final step, we conducted moderator analyses for a subset of outcomes with evidence of important heterogeneity between studies (i.e. *I*^2^ more than 75%) to investigate which study characteristics may account for a significant proportion of variation in effect sizes (Lipsey & Wilson, 2001). In order to include additional variables in our models, we had to further limit these analyses using the criteria described above (exposure and outcome measured the same way, same study design), as well as a cutoff of six or more studies available that fit these criteria. Note that in these analyses we were interested in identifying study-level (rather than individual-level) characteristics that may explain the heterogeneity in findings between studies. Drawing on existing literature, we identified four potential moderators of the relationship between education and sexual and reproductive health at the study level:

• Whether or not the study was published in a scientific journal (1) vs. a working paper (0), to address potential publication bias (Chan, Hróbjartsson, Haahr, Gøtzsche, & Altman, 2004);
• Whether the exposure was limited to a simple policy change (e.g. elimination of school fees, compulsory schooling) (0) or the policy change was combined with additional investments in the school infrastructure (e.g. building additional schools, hiring more teachers) (1), to account for possible effects of variations in school quality (World Bank, 2018);
• The lower bound of the age at which the outcome was measured, to account for potential censoring (continuous);
• A country-level indicator of the primary completion rate at the time the policy was enacted (continuous), to account for the national education environment (Caldwell, 1980; UNESCO Institute for Statistics (UIS), 2017). When data were not available for the year in which the policy was enacted, data from the closest year were used.

Data analyses were conducted in Stata version 14 using the command metareg (Harbord & Higgins, 2009).

## 3 Results

### 3.1 Included studies

**Fig. 1:**
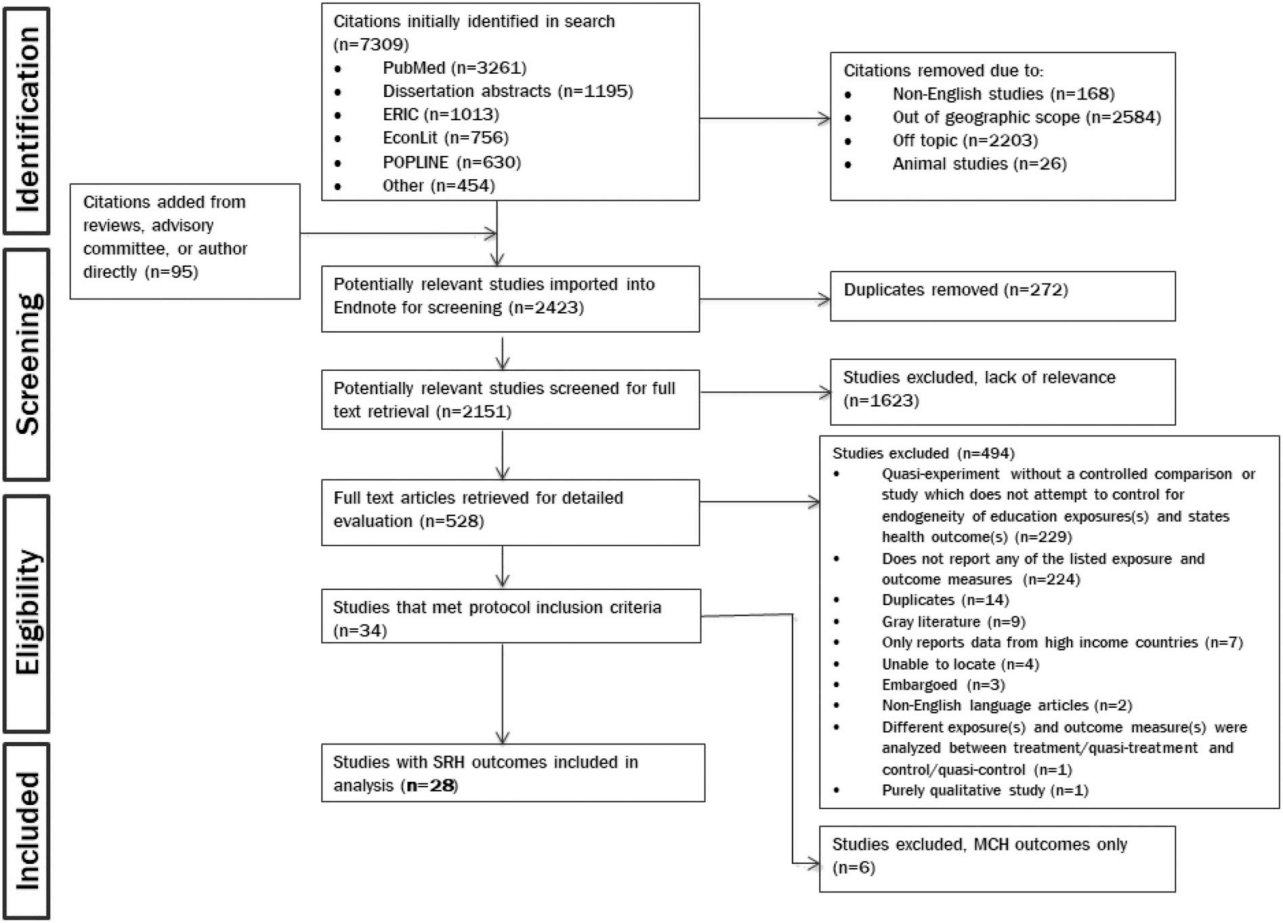
PRISMA flow chart. Fig. 1 outlines the search process for the entire review, including papers documenting the relationship between education and maternal and child health, which we do not report on in this paper.

Fig. 1 shows the PRISMA flow diagram, outlining the reasons for exclusion of papers throughout the screening process. In our initial search, 7309 papers were identified. An additional 102 papers were identified from reviews, or recommended by members of our advisory group or authors of the original group of studies identified. Of those, 5151 were irrelevant and eliminated. Abstracts of the remaining 2158 papers were screened for full text retrieval. During the abstract review process, we eliminated 1623 articles that did not meet our inclusion criteria, resulting in 535 articles for full text screening. During full text screening, an additional 494 studies were excluded because they did not meet our inclusion criteria, which was not apparent until we had reviewed these papers more thoroughly. This further exclusion resulted in 35 papers investigating the relationship between education and sexual and reproductive health (SRH) outcomes.^8^

### 3.2 Characteristics of included studies on sexual and reproductive health

Of the 35 studies that investigated the effects of education on SRH, the outcomes of interest were broken down as follows: 11 included age at first sex, 22 included age at first birth, 22 included age at marriage, 19 included parity/fertility, 16 included contraceptive use, and six included HIV status and other sexually transmitted infections (see Table 1). Most studies reported on numerous outcomes, including additional outcomes that were not the focus of this review. Although multiple studies investigated each outcome of interest, they often measured those outcomes using different indicators (e.g. probability of first birth by exact age x vs. age at first birth).

**Table 1:** Characteristics of included studies.

Outcomes	Number of studies
Age at first sex	11
Age at first marriage	22
Age at first pregnancy/birth	22
Contraceptive use	16
Parity/fertility	19
HIV/AIDS and other STIs	6
**Total Studies**	**35**

*Note:* Many studies examined more than one outcome, and are therefore counted multiple times in this table.

Identified papers were published between 2003 and 2018, with 20 published in 2015 or later. About half (19) were published in peer-reviewed scientific journals, while the rest were working papers (14) and dissertations (2). About two thirds of the papers (23) used data from sub-Saharan African countries, with repetition of countries, including Malawi (6), Ethiopia (3), Kenya (3), Uganda (3), Zimbabwe (2), and Ghana (2). The remaining papers used data from Turkey (3), Indonesia (2), China, Mexico, Nicaragua, Peru, Pakistan, Bangladesh, and Egypt.

All 35 of the identified papers solely measured the effects of access to school (e.g. school enrollment, grade attainment, or school attendance) on SRH outcomes. We did not identify any papers that measured the effects of learning (literacy or numeracy) on these outcomes. With a few exceptions, most studies did not assess whether there are threshold effects in the relationship between grade attainment and SRH outcomes, that is, whether the completion of primary or secondary school has a significant effect on health, beyond the effect of each additional year of schooling.

The majority of studies (27 out of 35) used quasi-experimental designs, exploiting natural experiments to address the endogeneity in the relationship between education and SRH. As an example, several of the papers using data from Malawi (Behrman, 2015a, Behrman, 2015b; Grant, 2015a, Grant, 2015b; Makate & Makate, 2016) exploit the passage of a free primary education policy in 1994 as an exogenous source of variation in exposure to education, which, they report, led to significant increases in grade attainment among those exposed. As a result, these authors were able to statistically address the endogeneity in the relationship between education and health outcomes. Of the papers drawing on natural experiments, 20 used Demographic and Health Survey data. The eight remaining studies used data from program evaluations, seven of which were randomized controlled trials (RCTs), and one of which was quasi-experimental (Alam, Javier, & Ximena, 2011). Four of the papers (Baird, Chirwa, McIntosh, & Berk, 2010; Baird, Garfein, McIntosh, & Ozler, 2012; Baird, McIntosh, & Özler, 2011) used data from the same RCT in Malawi but assessed different outcomes in slightly different samples. In most of the models from RCTs, with an exception of those reported by Duflo and colleagues (Duflo, Dupas, & Kremer, 2017), the authors did not isolate the direct effect of increased education (e.g. grade attainment) on health outcomes, but rather examined the effects of the intervention itself (e.g. conditional cash transfers) on health outcomes. In these cases, the interventions improved grade attainment, and also improved SRH, but we do not know how much of the improvement in SRH is attributable to improvements in education versus other effects of the intervention (e.g. increasing household resources). We indicate these cases in all results tables.

Not surprisingly given our strict inclusion criteria, most (24) included studies were rated as high quality using our study quality assessment tool. Ten were rated as “medium” quality (score of 3), and one was rated as “low” quality (score of 1–2). These ratings are more a reflection of the information reported by study authors than the rigor of the study designs or analyses. We did not identify enough studies assessing each outcome to conduct statistical subgroup analyses based on study quality, but it appears that limiting our results to only high-quality studies would not substantially change the distribution of results within each outcome. Therefore, we do not report results by level of study quality.

The identified papers investigated the relationship between increased grade attainment and each outcome in multiple ways. We present only the results for outcome measures for which we were able to run meta-regressions (i.e. at least three independent models measured the exposure and outcome in the same way). Additional results are presented in supplementary tables. Since only four of the papers identified reported results for men or boys, we focus our presentation of results on models for women and girls. A summary table of results on men and boys is included in our supplementary files (Table S8).

### 3.3 Age at first sex

Our search identified 11 papers investigating the effect of education on age at first sex. All papers except one (Barham, Macours, & Maluccio, 2018) used data from sub-Saharan Africa. Of the papers using data from RCTs, all examined the effects of the interventions themselves (conditional cash transfers) on age at first sex, rather than isolating the direct effect of improved education on age at first sex. The two papers by Baird and colleagues (Baird et al., 2010, 2012) draw on overlapping samples from the same RCT in Malawi, and the results from each are stratified by baseline enrollment status (dropouts vs. school girls) given the authors’ assumption that the effects of the intervention on SRH outcomes are likely to differ between groups.

Given the limited number of studies measuring the exposure and outcome the same way, we only present forest plots and meta-regression results for those assessing the effect of increases in grade attainment on age at first sex, measured continuously (Table 2). The estimated mean effect size of 0.03 was not significantly different from zero (p = 0.11). The *Q* statistic (0.81, p > 0.10) indicates only borderline significant heterogeneity, possibly due to the small number of studies. As a result, the *I*^2^ value was not calculated. Given the lack of evidence of heterogeneity and small sample size, we did not conduct moderator analyses for this relationship.

**Table 2:**
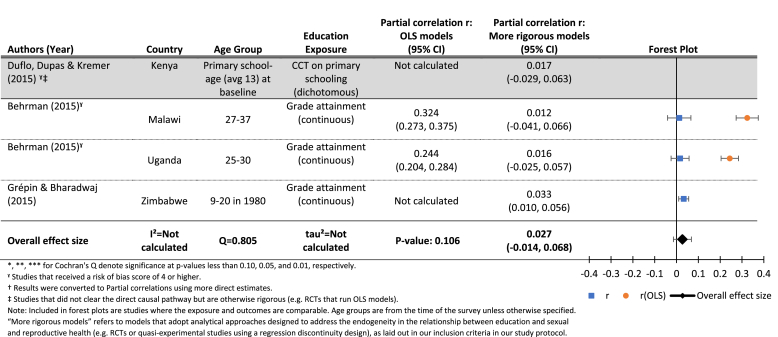
Results for the effects of education (grade attainment) on age at first sex (continuous).

Included citations: Duflo et al. (2015), Behrman, 2015a, Behrman, 2015b; Grépin and Bharadwaj (2015).

One of the papers investigating the effect of grade attainment on age at first sex included analyses from two countries, and presented results from both naïve OLS models and more rigorous models accounting for shared underlying causes of education and the timing of first sex (Behrman, 2015a, Behrman, 2015b). In both countries, the results from the OLS models indicated a statistically significant positive effect of education on age at first sex, but the estimate was attenuated and no longer significant in the more rigorous models.

**Fig. 2:**
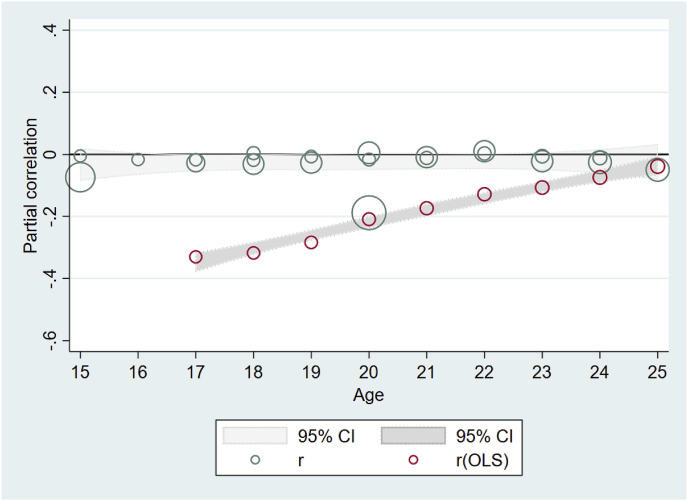
Estimated effects (and 95% confidence intervals) of increased exposure to formal schooling on the probability of first sex by age X. **Notes**: Each circle represents an estimate from a study included in this review. The red circles represent estimates from OLS models that do not account for the shared determinants of exposure to schooling and age at first sex. The green circles are estimates from models that address that endogeneity. The size of the circles reflects the standard errors of each estimate, while the fitted lines and 95% confidence intervals show the estimated trend in the relationship between exposure to schooling and age at first sex, by age. (For interpretation of the references to colour in this figure legend, the reader is referred to the web version of this article.)

Although we were unable to generate meta-analysis results for the effects of grade attainment on the probability of first sex by exact age x, we display results from those models in Fig. 2, which shows estimated partial correlations from OLS and more rigorous models, along with 95% confidence intervals, by age. We present the results this way in order to show two patterns: 1) the differences between estimates from OLS and more rigorous models, and 2) the changing estimates from both models by age. Fig. 2 shows clearly that, especially at younger ages, OLS models indicate a significant and substantial effect of increased grade attainment on delaying age at first sex. However, the same data, when analyzed using models that account for the selectivity of girls who stay in school longer, show few significant effects, all of which are much smaller than the OLS models.

### 3.4 Age at marriage/cohabitation

Our search identified 22 papers investigating the effect of education on age at marriage/cohabitation for girls or women. Fourteen studies exploited natural experiments that increased exposure to education, seven used data from RCTs of interventions designed to improve education outcomes (including three from the same RCT), and one used data from a quasi-experimental program evaluation. Of the seven papers using data from RCTs, six examined the effects of the interventions themselves (conditional cash transfers) on age at marriage, rather than isolating the direct effect of improved education on age at marriage (exception was Duflo et al., 2017).

The authors took multiple approaches to measuring the outcome; Table 3.1, Table 3.2 present the results, including forest plots, for the two approaches with a sufficient number of papers to conduct meta-analyses: age at marriage measured as a continuous (Table 3.1) and a dichotomous (Table 3.2) variable. Additional results are presented in Supplementary Tables S3.1-S3.2. Overall results are mixed and show inconsistent evidence in support of an effect of increased exposure to school on later age at marriage.^9^

**Table 3.1:**
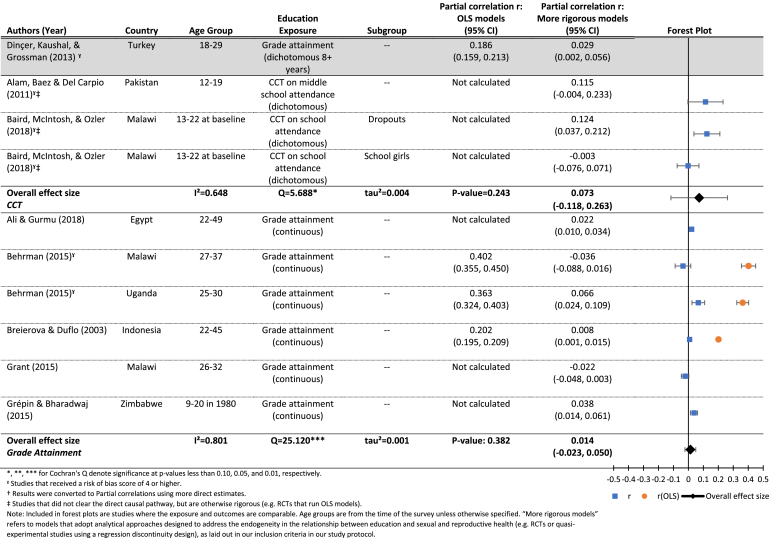
Results for the effects of education (grade attainment) on age at marriage (continuous).

Included citations: (Dinçer, Kaushal, and Grossman (2014), Alam et al., 2011a, Alam et al., 2011b, Baird et al. (2018), Ali & Gurmu (2016), Behrman, 2015a, Behrman, 2015b; Breierova and Duflo (2004); Grant, 2015a, Grant, 2015b; Grépin and Bharadwaj (2015)).

**Table 3.2:**
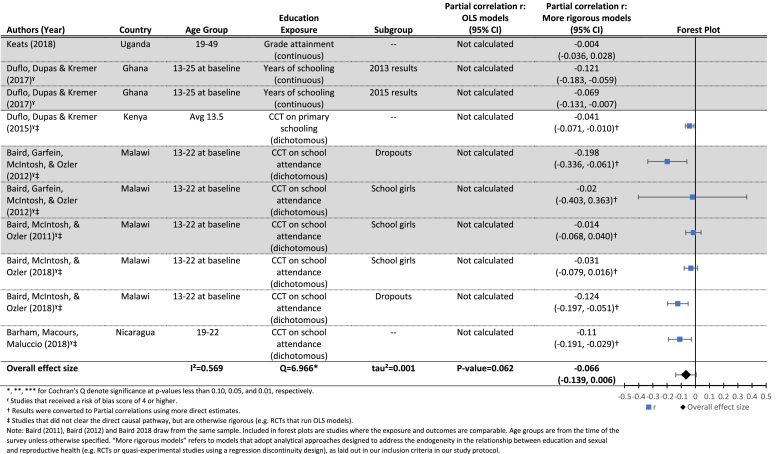
Results for the effects of education (grade attainment) on the probability of having ever been married (dichotomous).

Included citations: (Keats (2018); Duflo et al. (2017); Duflo et al. (2017); Duflo et al. (2015); Baird et al. (2012); Baird et al. (2011); Baird et al. (2018)).

Table 3.1 breaks down the results for RCTs testing the effect of conditional cash transfers on age at marriage (upper panel), and quasi-experimental studies testing the effect of each additional year of education on age at marriage (lower panel). The cash transfer results are null overall, with a mean effect of 0.07 (p = 0.24). The Q statistic (5.69, p < 0.10) provides evidence of heterogeneity between studies, and the *I*^2^ statistic indicated that 65% of the variation between studies is due to the studies themselves. We were unable to conduct moderator analysis due to the insufficient number of studies.

The estimated mean effect of each additional year of grade attainment on age at marriage (lower panel of Table 3.1) is smaller than that from the CCT studies, and not significantly different from zero (r = 0.01, p = 0.38). Again, the *Q* statistic (25.12, p < 0.05) points to significant heterogeneity between studies, and the *I*^2^ indicates that 80% of the variation between studies is due to the studies themselves, rather than sampling error. In this case, we were able to conduct moderator analyses in an attempt to understand the sources of the variation between studies. However, none of the four variables included were significant moderators of the estimated relationship between grade attainment and age at marriage at the study level, possibly due to the small number of studies included in the analyses (see Table 8) (Hempel et al., 2013).

**Fig. 3:**
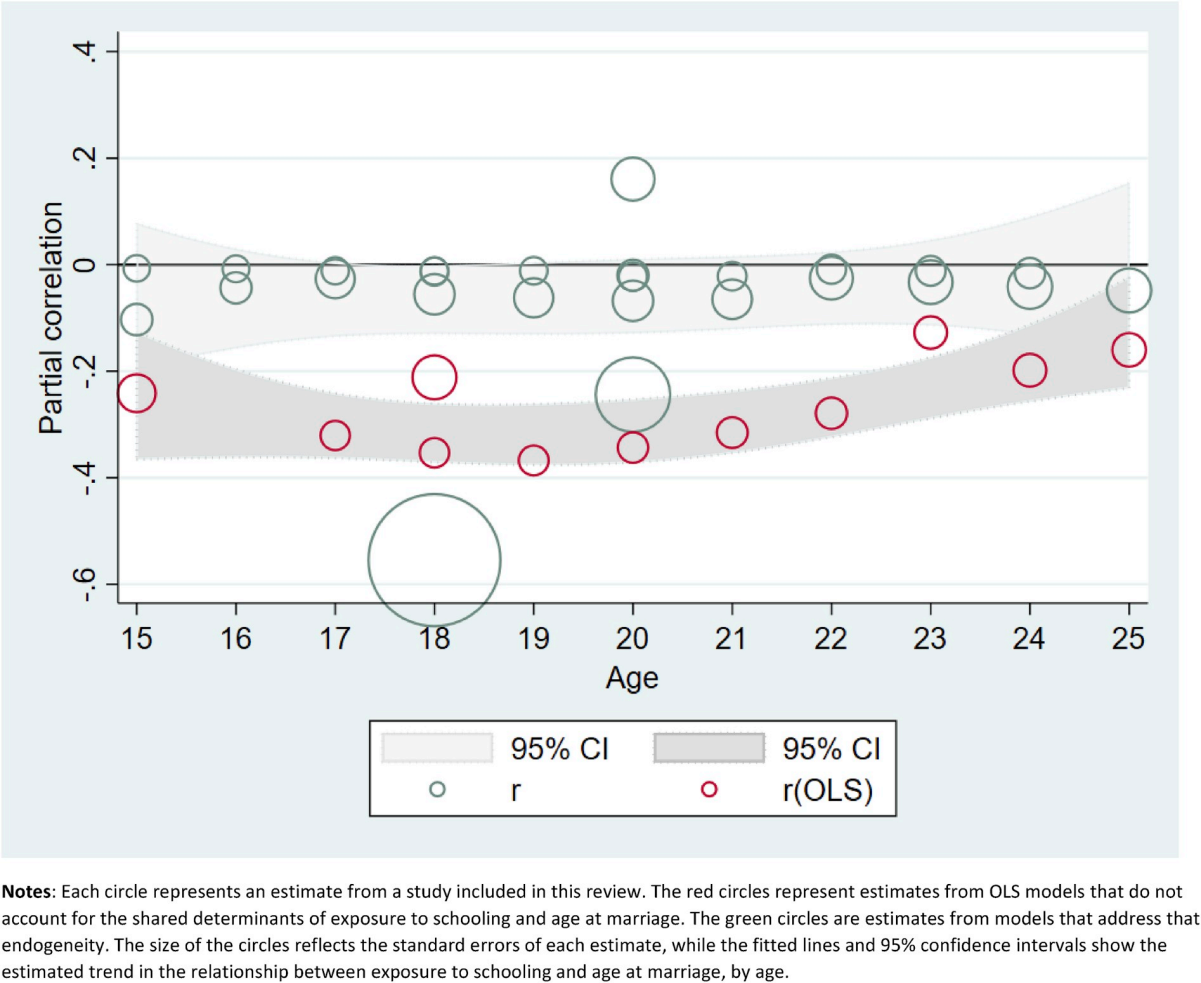
Estimated effects (and 95% confidence intervals) of increased exposure to formal schooling on the probability of marriage by age X. **Notes**: Each circle represents an estimate from a study included in this review. The red circles represent estimates from OLS models that do not account for the shared determinants of exposure to schooling and age at marriage. The green circles are estimates from models that address that endogeneity. The size of the circles reflects the standard errors of each estimate, while the fitted lines and 95% confidence intervals show the estimated trend in the relationship between exposure to schooling and age at marriage, by age. (For interpretation of the references to colour in this figure legend, the reader is referred to the web version of this article.)

Table 3.1 and Fig. 3 compare results from OLS and more rigorous models controlling for endogeneity. In the case of Behrman, 2015a, Behrman, 2015b analyses in Malawi and Uganda (Table 3.1), the OLS estimates for both countries were moderately large in magnitude in the expected direction, indicating that more education led to significantly older age at marriage. The more rigorous models showed a much smaller but statistically significant effect in Uganda, but a null result in Malawi. In comparing the OLS and more rigorous models for marriage by exact age x (Fig. 3), the estimates are attenuated, and often no longer statistically significant, in the more rigorous models.

Table 3.2 presents results for the effects of increased grade attainment (upper panel) or a CCT improving grade attainment (lower panel), on the probability of having ever been married at follow-up. The mean effect in the lower panel is borderline significant (r = −0.07, p = 0.06), indicating a small effect of CCTs conditional on school attendance (which lead to increased attainment) on the probability of having ever been married at follow-up. Results for the effects of grade attainment on the probability of marriage are similar, although we did not identify a sufficient number of studies to estimate a mean effect size.

### 3.5 Age at first pregnancy/birth

Our search identified 22 papers investigating the effect of education on age at first pregnancy/birth. The papers investigating this relationship overlap almost entirely with those investigating the effects of education on age at marriage. Table 4.1, Table 4.2 separate the results by two of the approaches authors took to measuring this outcome: age at first birth measured as a continuous variable (Table 4.1), and the probability of having ever been pregnant/given birth by follow-up (Table 4.2). Some papers included multiple models and are therefore listed more than once.

**Table 4.1:**
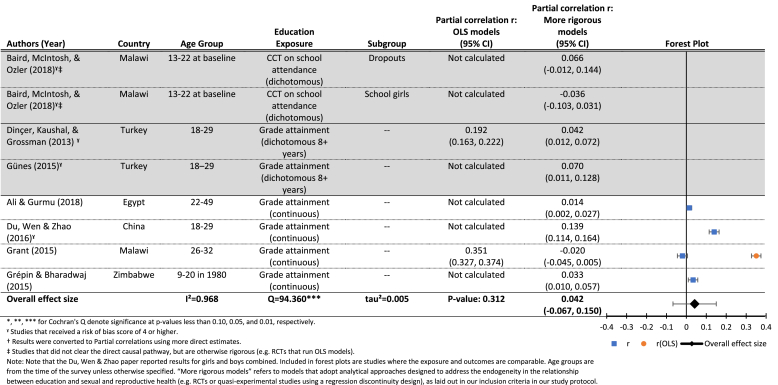
Results for the effects of education (grade attainment) on age at first birth (continuous).

Included citations: (Baird et al. (2018), Dinçer et al. (2014), Güneş (2015), Ali & Gurmu (2016), Du, Wen & Zhao (2016); Grant, 2015a, Grant, 2015b; Grépin and Bharadwaj (2015))

**Table 4.2:**
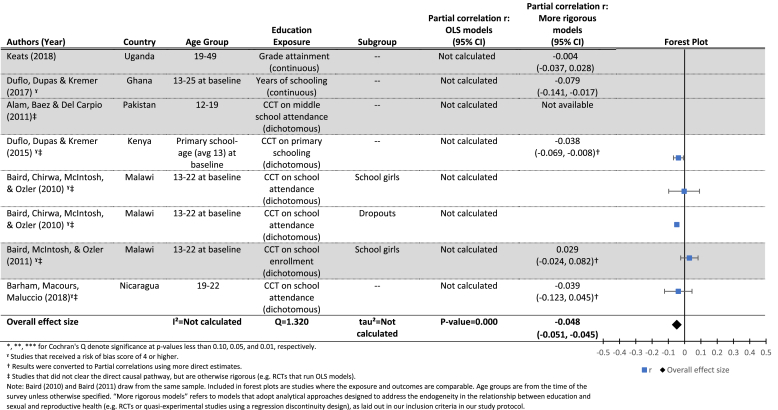
Results for the effects of education (grade attainment) on the probability of having ever been pregnant/given birth (dichotomous).

Included citations: (Keats (2018); Duflo et al. (2017); Alam et al., 2011a, Alam et al., 2011b; Duflo et al. (2015); Baird et al. (2010); Baird et al. (2011); Barham et al. (2018)).

Table 4.1 presents results on the effects of three different exposures, all of which are measuring increased exposure to schooling in some way: cash transfer programs conditional on school attendance, a dichotomous measure of grade attainment (8 or more years), and a continuous measure of grade attainment. Results for the effects of CCTs on age at first birth are null (top panel), as is the mean effect of one additional year of schooling (lower panel), although the effects of 8 or more years of schooling are small but significantly different from zero (middle panel).We were only able to estimate a mean effect for the continuous measure of grade attainment (r = 0.04, p > 0.10), and again found significant heterogeneity between studies (Q = 94.4, p < 0.01), and found that nearly all of the variation between estimates was due to the studies themselves (*I*^*2*^ = 0.97). We were unable to run moderator analyses due to an insufficient number of studies. We also see again that the one study (Grant, 2015a, Grant, 2015b) that estimated both OLS and more rigorous models, found strong significant effects of grade attainment on delayed childbearing, which were wiped out after addressing endogeneity.

Overall the results on the effects of increased grade attainment on the probability of having ever given birth at follow-up indicate a small but statistically significant effect (see Table 4.2). After combining those assessing a similar exposure (cash transfer conditional on school attendance), we find a small negative estimated mean effect of grade attainment on age at first birth (0.05, p < 0.01). We did not find evidence of significant heterogeneity between studies (Q = 1.3, p > 0.10), and we were unable to conduct moderator analysis due to small sample size.

**Fig. 4:**
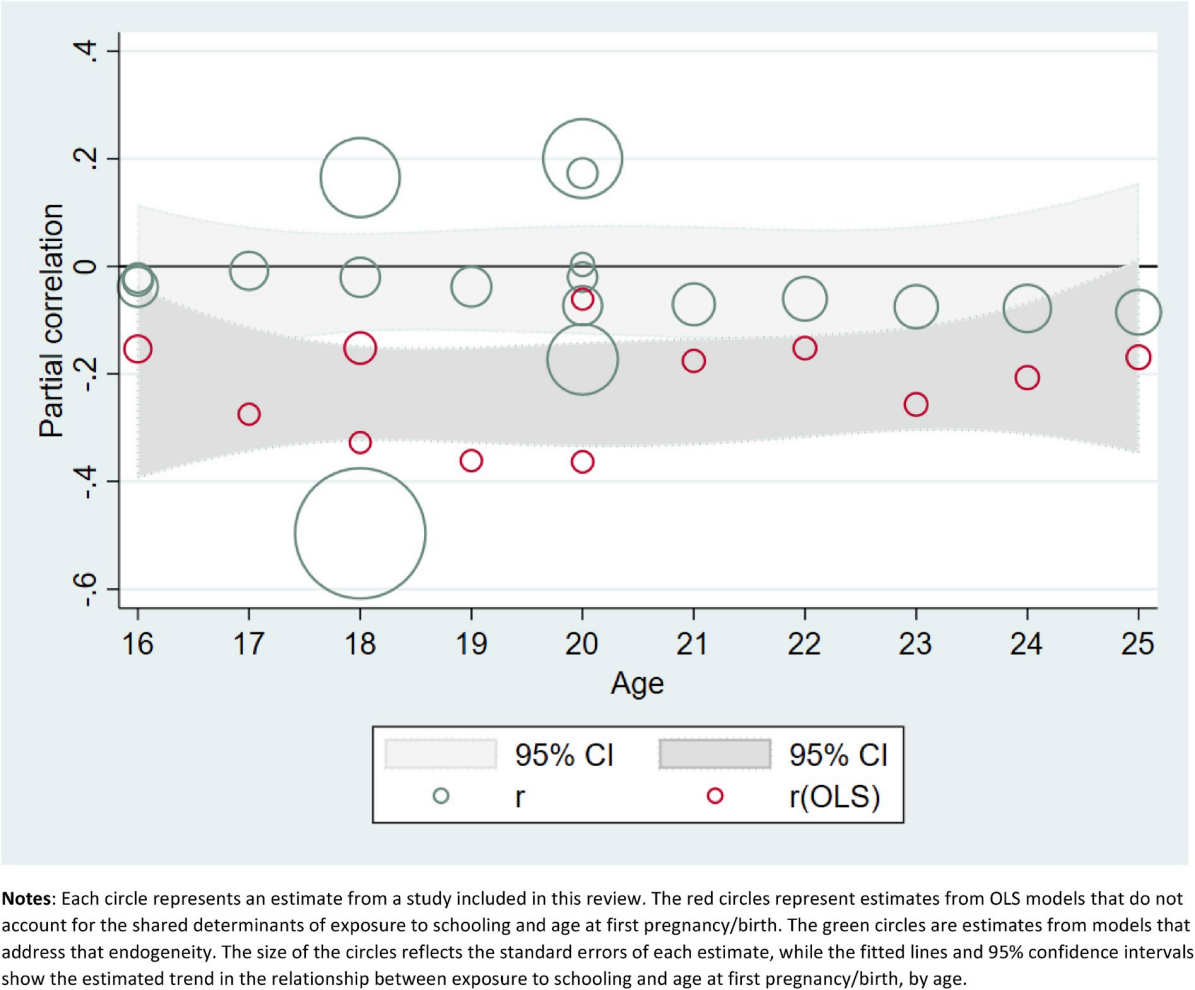
Estimated effects (and 95% confidence intervals) of increased exposure to formal schooling on the probability of first pregnancy/birth by age X. **Notes**: Each circle represents an estimate from a study included in this review. The red circles represent estimates from OLS models that do not account for the shared determinants of exposure to schooling and age at first pregnancy/birth. The green circles are estimates from models that address that endogeneity. The size of the circles reflects the standard errors of each estimate, while the fitted lines and 95% confidence intervals show the estimated trend in the relationship between exposure to schooling and age at first pregnancy/birth, by age. (For interpretation of the references to colour in this figure legend, the reader is referred to the web version of this article.)

Fig. 4 shows the results on the effects of increased grade attainment on the probability of having a first pregnancy or birth by exact age X. Again, we see that OLS estimates of this relationship are substantially attenuated in the more rigorous models. However, similarly to Table 4.2, this figure shows small but statistically significant effects of increased grade attainment on age at first birth, especially among thoseover age 18.

### 3.6 Contraceptive use

Our search identified 16 papers investigating the effect of education on contraceptive use. Of the three papers using data from RCTs, all examined the effects of the interventions themselves (conditional cash transfers) on age at first pregnancy/birth, rather than isolating the direct effect of improved education on age at first pregnancy/birth. Most of the 16 papers investigating the relationship between education and contraceptive use specified the outcome as either current contraceptive use (Table 5.1) or ever use of modern contraception (Table 5.2). Other specifications of contraceptive use, such as intercourse without a condom in the previous 12 months (Baird et al., 2012), are included in the supplementary tables.

**Table 5.1:**
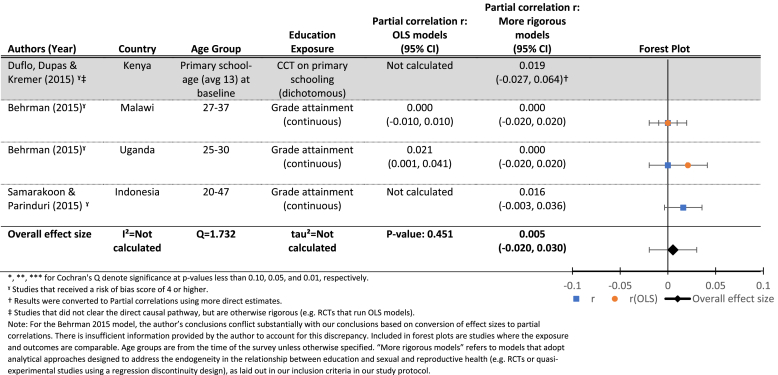
Currently using modern contraception (dichotomous).

Included citations: (Duflo et al. (2015), Behrman, 2015a, Behrman, 2015b, Samarakoon and Parinduri (2015)).

**Table 5.2:**
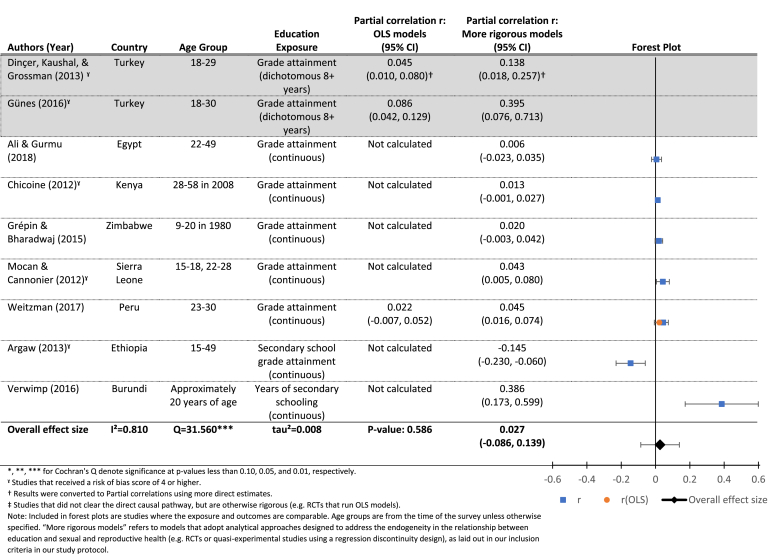
. Ever used modern contraception (dichotomous).

Included citations: (Dinçer et al. (2014), Güneş (2016), Ali & Gurmu (2016), Chicoine (2012); Grépin and Bharadwaj (2015); Mocan and Cannonier (2012); Weitzman (2017); Argaw (2013); Verwimp (2016)).

Table 5.1 shows null results for all studies assessing the effects of increased exposure to schooling on current use of modern contraception. Findings do not differ systematically in one direction when comparing OLS and more rigorous models, when available. The estimated mean effect (r = 0.005, p > 0.10) reflects these findings, and we find no indication of significant heterogeneity between studies (Q = 1.7, p > 0.10). Table 5.2 show similarly null results regarding the effects of increased exposure to schooling on lifetime use of modern contraception.

We find a small and statistically nonsignificant estimated mean effect of increased grade attainment (continuous) on ever use of modern contraception (r = 0.03, p = 0.59, with evidence of significant heterogeneity between studies (Q = 31.60, p < 0.01), which is largely attributable to differences between the studies themselves (*I*^2^ = 0.81). However, no significant relationships between hypothesized moderators and the effects of grade attainment on ever use of contraception were observed, possibly due to lack of power (see Table 8).

### 3.7 Parity/fertility

**Fig. 5:**
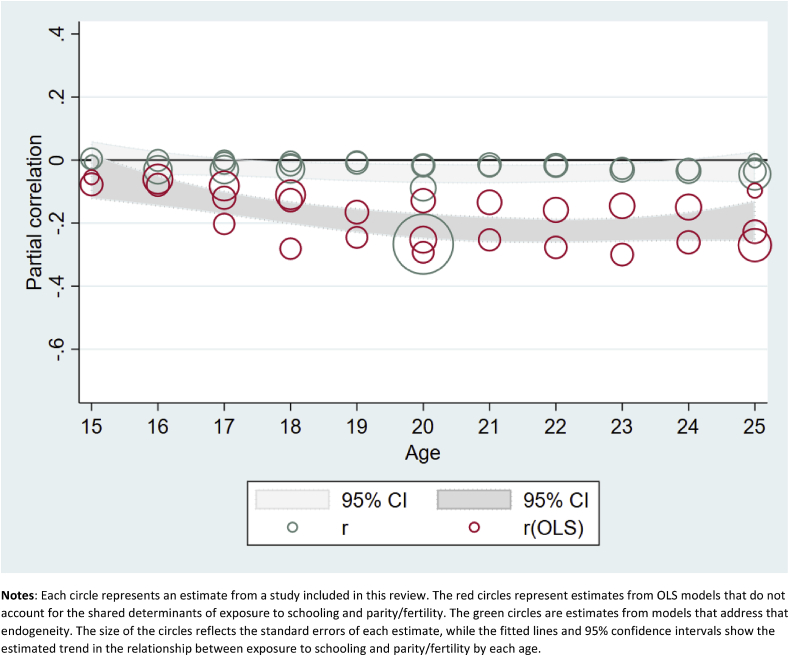
Estimated effects (and 95% confidence intervals) of increased exposure to formal schooling on the parity/fertility by age X. **Notes**: Each circle represents an estimate from a study included in this review. The red circles represent estimates from OLS models that do not account for the shared determinants of exposure to schooling and parity/fertility. The green circles are estimates from models that address that endogeneity. The size of the circles reflects the standard errors of each estimate, while the fitted lines and 95% confidence intervals show the estimated trend in the relationship between exposure to schooling and parity/fertility by each age. (For interpretation of the references to colour in this figure legend, the reader is referred to the web version of this article.)

Our search identified 19 papers investigating the effect of education on parity/fertility. The 19 papers investigating this relationship specified the outcome in two different ways: a continuous measure of total pregnancies/births by the time of the survey (shown in Table 6), and total pregnancies/births by exact age x (shown in Fig. 5).^10^ Given the ages at follow-up, few studies measured completed fertility for the entire sample. Therefore, conceptually there is overlap between the results on the effects of education on age at first birth, and those for parity/fertility. However, the results presented in Table 6 are drawn from different models than those presented in Table 4.1, Table 4.2.

**Table 6:**
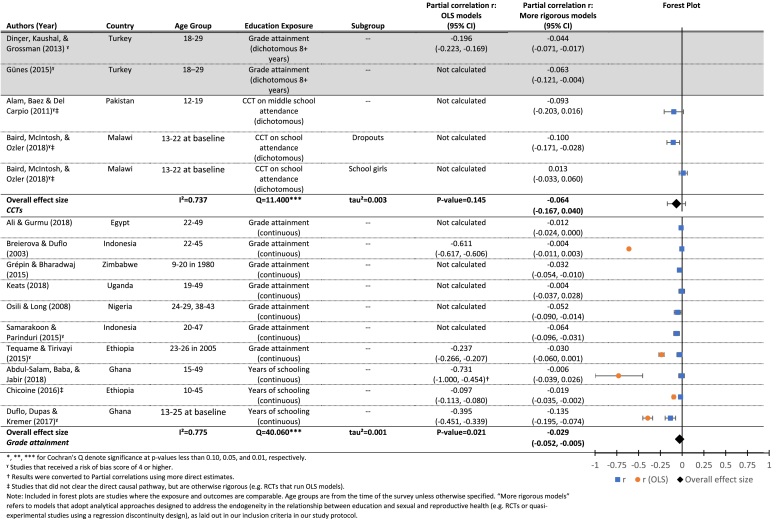
Results for the effects of education (grade attainment) on parity/fertility, measured as total pregnancies/births (continuous).

Included citations: (Dinçer et al. (2014), Güneş (2015), Alam et al., 2011a, Alam et al., 2011b, Baird et al. (2018), Ali & Gurmu (2016), Breierova and Duflo (2004); Grépin and Bharadwaj (2015); Keats (2018); Osili and Long (2008); Samarakoon and Parinduri (2015); Tequame and Tirivayi (2015); Abdul-Salam, Baba, and Jabir (2018); Chicoine (2016); Duflo et al. (2017)).

Many of the results on total pregnancies/births by exact age x (Fig. 5) are small but statistically significant, indicating delaying or spacing of childbearing. OLS models again showed much more substantial effects in the expected direction than more rigorous models addressing endogeneity. Table 6 includes three panels, showing the exposure (grade attainment) measured in three different ways: as a dichotomous variable measuring eight or more years of schooling (top panel), as a cash transfer program conditional on school attendance (middle panel), and as a continuous increase in grade attainment (lower panel). All three show small, sometimes statistically significant, effects, indicating that those with higher grade attainment may have somewhat fewer pregnancies or births. Meta-regression results from the middle panel (CCTs) indicate a small and nonsignificant mean effect of increased grade attainment on total pregnancies/births (r = −0.06, p = 0.15). We also find evidence of significant heterogeneity between studies (Q = 11.4, p < 0.01), which is largely attributable to differences between the studies themselves (*I*^2^ = 0.74). In the lower panel, the small estimated effect of grade attainment on parity/fertility is significant (r = −0.03, p < 0.05) and again we find evidence of significant heterogeneity (Q = 40.10, p < 0.01) attributable to the studies themselves (*I*^*2*^ = 0.78), but none of the moderators explain this heterogeneity, possibly due to lack of power (see Table 8).

### 3.8 HIV/AIDS and other STIs

Our search identified six papers investigating the effect of education on HIV status and other STIs, all of which used data from sub-Saharan Africa. The identified papers assessed three different outcomes: HIV status, HSV-2 status, and Syphilis status. Table 7.1, Table 7.2 show the results for HIV status and HSV-2 status, respectively.

**Table 7.1:**
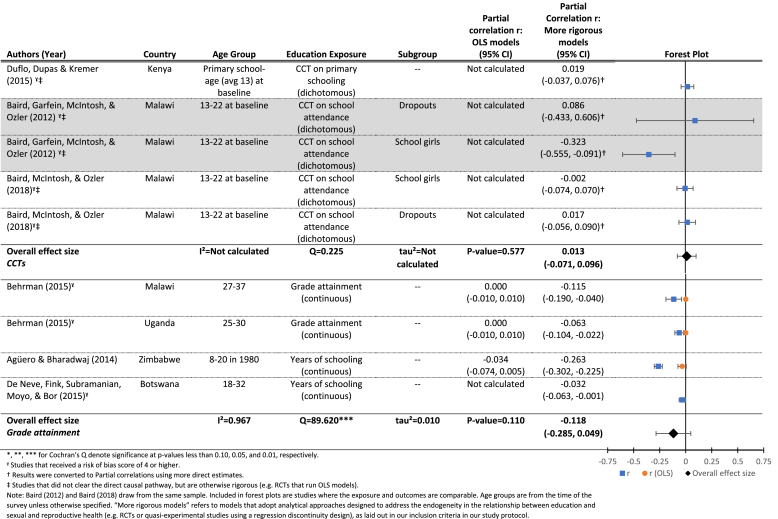
Results for the effects of education (grade attainment) on HIV status (dichotomous).

Included citations: (Duflo et al. (2015); Baird et al. (2012); Baird et al. (2018); Behrman, 2015a, Behrman, 2015b; Agüero and Bharadwaj (2014); De Neve et al., 2015a, De Neve et al., 2015b)

**Table 7.2:**
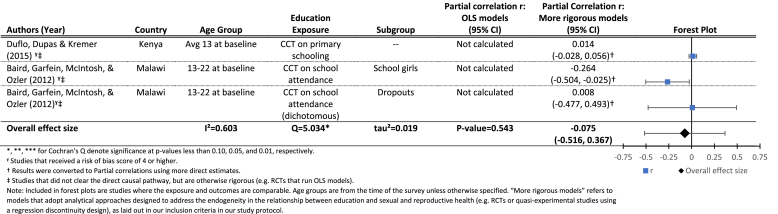
. Results for the effects of education (grade attainment) on HSV-2 status (dichotomous).

Included citations: (Duflo et al. (2015); Baird et al. (2012)).

Table 7.1 shows the effects of two different types of exposures on HIV status: cash transfers, conditional on school attendance (upper panel), and increased grade attainment, measured continuously (lower panel). In both cases, the mean effects do not differ significantly from zero. That is, overall we find limited evidence that increased exposure to schooling reduces the likelihood of being HIV positive. In the upper panel (CCTs), we find an estimated mean effect of 0.01 (p = 0.58), and we find no evidence for significant variation between studies (Q = 0.23, p > 0.10). Note that this estimate excludes the results from the Baird et al. (2012) paper, since the more recent paper (Baird, McIntosh, & Özler, 2018) presents data from the same sample. Also, Duflo, Dupas, and Kremer (2015) note that, given lower than expected HIV prevalence in the sample, the study was not sufficiently powered to estimate differences in HIV status due to the intervention (Duflo et al., 2015).

In the lower panel (grade attainment), we find somewhat more promising results (r = −0.12, p = 0.11). Although the mean effect is on the border of statistical significance at the 0.10 alpha level, each of the included studies reports a statistically significant effect of increased exposure to education on lower probability of being HIV positive at follow-up.^11^ We find evidence of significant heterogeneity between studies (Q = 89.60, p < 0.01), most of which is attributable to differences between studies (*I*^2^ = 0.97). The sample of studies identified was insufficient to conduct moderator analyses.

Table 7.2 presents the results for the effects of cash transfers, conditional on school enrollment/attendance on HSV-2 status. We find a null mean effect (r = −0.08, p = 0.54), as well as evidence for significant heterogeneity between studies (Q = 5.0, p < 0.10), much of which is attributable to differences between studies (*I*^*2*^ = 0.60).

### 3.9 Evidence for mechanisms underlying causal relationships

This review was designed to identify studies that estimate the causal relationship between education and sexual and reproductive health. However, many of the identified studies also discussed, and tested empirically, the mechanisms underlying those relationships. While we did not conduct a systematic review of all literature documenting these mechanisms, we provide a summary of the findings included in identified papers to contextualize the results of the larger review. A more systematic review of these mechanisms is warranted, as much additional evidence undoubtedly exists that is not captured here.

**Fig. 6:**
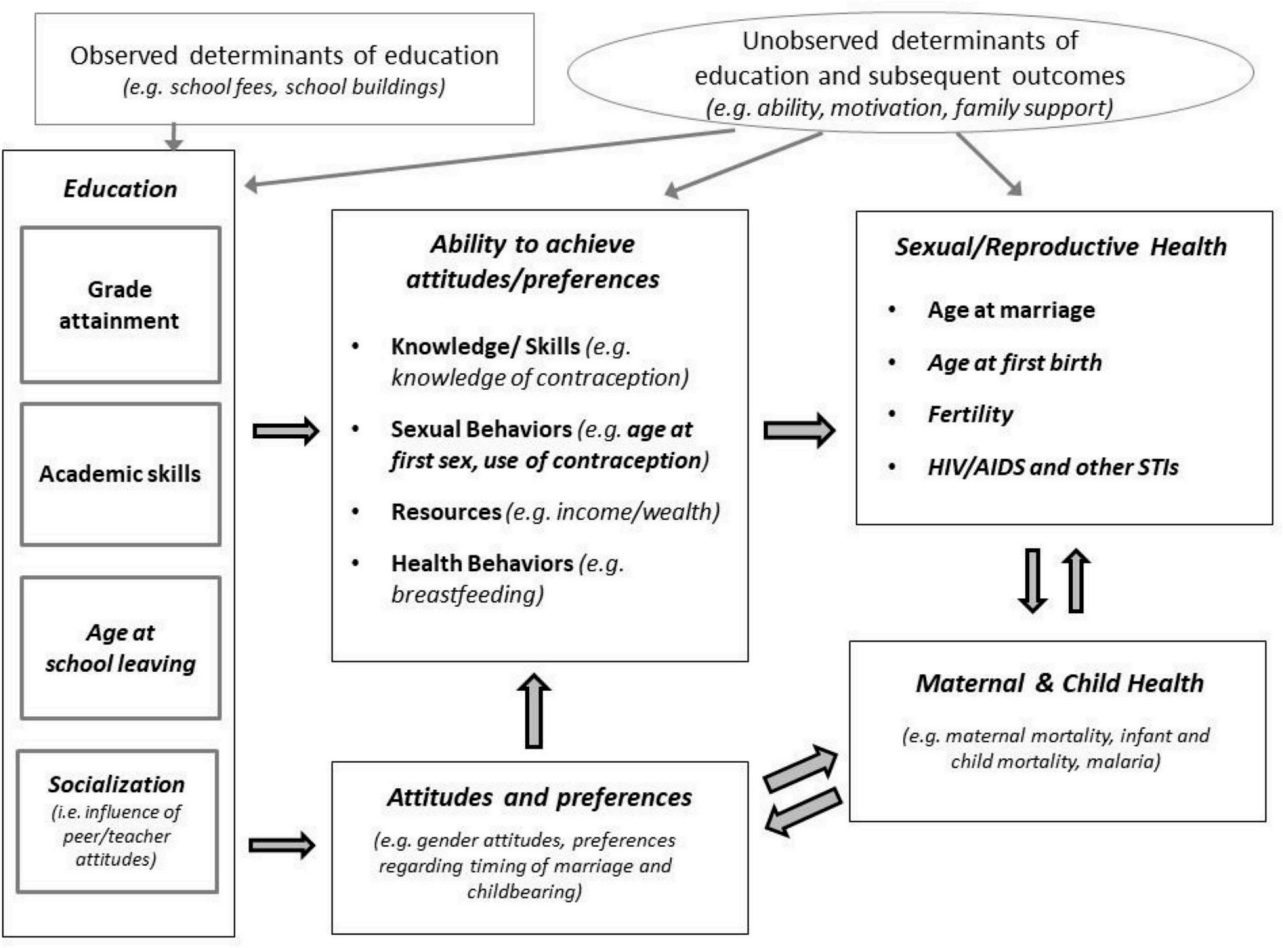
Conceptual framework showing hypothesized mechanisms linking education and sexual and reproductive health.

As described, theoretical literature on the links between education and SRH highlights numerous potential pathways, which roughly fall into one of two groups: the effect of education on changing preferences, and the effect of education on changing ability to achieve those preferences. Fig. 6 provides an overview of hypothesized mechanisms linking education and SRH based on the theoretical literature. Note that, although papers included in our review provide causal evidence of the relationships between education and SRH, many did not adopt equally rigorous approaches to testing the mechanisms linking exposures and intermediate variables. Therefore, not all of the results on mechanisms can be interpreted causally.

Table 9 includes a row for each of the main hypothesized pathways linking education and SRH in the theoretical and empirical literature, as described previously. The columns show the papers that found empirical evidence in support of that pathway, and those that tested the pathway and did not find support in their data. Note that not all papers tested mechanisms, and those that did often only tested a subset of hypothesized pathways. Most analyses looked only at whether education was linked with the intervening variable (e.g. fertility preferences), and did not test whether the intervening variable explained all or part of the relationship between education and the outcome of interest. Last, most analyses of pathways focused on fertility as an outcome, although, as noted, authors did not test the full pathway from education to fertility.

**Table 8:** Summary of results from meta-analyses.

Outcome	Type of Study	Mean effect size	95% confidence interval	Homogeneity Analyses			Moderator Analyses			
				τ^2^	*I*^2^	Q statistic	Journal article	Policy + investment	Minimum age at follow-up	% primary completion, national
Age at first sex (continuous)	Quasi-experiments	0.027 (p > 0.10)	(-0.014, 0.068)	*Not calculated*	*Not calculated*	0.805 (p > 0.10)	–	–	–	–
Age at first marriage (continuous)	Quasi-experiments	0.014 (p > 0.10)	(-0.023, 0.050)	0.001	0.801	25.120 (p < 0.01)	0.332 (p > 0.10)	−0.190 (p > 0.10)	−0.039 (p > 0.10)	−0.025 (p > 0.10)
Age at first marriage (continuous)	CCTs	0.073 (p > 0.10)	(-0.118, 0.263)	0.004	0.648	5.688 (p < 0.10)	–	–	–	–
Ever married/cohabited (dichotomous)	CCTs (using results from Baird et al. (2018)	−0.066 (p < 0.10)	(-0.139, 0.006)	0.001	0.569	6.966 (p < 0.10)	–	–	–	–
Age at first birth (continuous)	Quasi-experiments	0.042 (p > 0.10)	(-0.067, 0.150)	0.005	0.968	94.360 (p < 0.01)	–	–	–	–
Ever pregnant (dichotomous)	CCTs (using results from Baird et al. (2010)	−0.048 (p < 0.01)	(-0.051, −0.045)	*Not calculated*	*Not calculated*	1.320 (p > 0.10)	–	–	–	–
Currently using contraception (dichotomous)	Quasi-experiments	0.005 (p > 0.10)	(-0.020, 0.030)	*Not calculated*	*Not calculated*	1.732 (p > 0.10)	–	–	–	–
Ever used contraception (dichotomous)	Quasi-experiments	0.027 (p > 0.10)	(-0.086, 0.139)	0.008	0.810	31.560 (p < 0.01)	−0.004 (p > 0.10)	0.023 (p > 0.10)	0.0003 (p > 0.10)	0.002 (p > 0.10)
Total births (continuous)	Quasi-experiments	−0.029 (p < 0.05)	(-0.052, −0.005)	0.001	0.775	40.060 (p < 0.01)	0.015 (p > 0.10)	0.006 (p > 0.10)	0.001 (p > 0.10)	−0.0004 (p > 0.10)
Total births (continuous)	CCTs	−0.064 (p > 0.10)	(-0.167, 0.040)	0.003	0.737	11.400 (p < 0.01)	–	–	–	–
HIV positive status (dichotomous)	Quasi-experiments	−0.118 (p > 0.10)	(-0.285, 0.049)	0.010	0.967	89.620 (p < 0.01)	–	–	–	–
HIV positive status (dichotomous)	CCTs (using results from Baird et al. (2018)	0.013 (p > 0.10)	(-0.071, 0.096)	*Not calculated*	*Not calculated*	0.225 (p > 0.10)	–	–	–	–
HSV-2 positive status (dichotomous)	CCTs	−0.075 (p > 0.10)	(-0.516, 0.367)	0.019	0.603	5.034 (p < 0.10)	–	–	–	–

Note: Table only shows outcomes for which meta-regressions were conducted.

**Table 9:** Evidence in support of hypothesized mechanisms linking education and sexual and reproductive health.

	Support for pathway	No support for pathway (null)
*Changing attitudes and preferences*		

Fertility preferences: *Evidence for effects of increased grade attainment on lower desired fertility*	For women: • Chicoine, 2012 (Kenya) • Chicoine, 2016 (Ethiopia) • Grépin & Bharadwaj, 2015 (Zimbabwe) • Keats, 2018 (Uganda) • Mocan & Cannonier, 2012 (Sierra Leone) • Verwimp, 2016 (Burundi) For men: *None*	For women: • Ali & Gurmu, 2016 (Egypt) • Argaw, 2013 (Ethiopia) • Duflo et al., 2017 (Ghana) • Samarakoon & Parinduri, 2015 (Indonesia) For men: • Duflo et al., 2017 (Ghana) • Keats, 2016 (Uganda, male partners of women with increased education) • Mocan & Cannonier, 2012 (Sierra Leone)

Gender attitudes: *Evidence for effects of increased grade attainment on more equitable gender attitudes (measured differently across studies)*	For women: • Mocan & Cannonier, 2012 (Sierra Leone, attitudes toward women refusing sex with husband, disapproval of wife beating) • Tequame & Tirivayi, 2015 (Ethiopia, attitudes toward decisions about own healthcare in urban sample) For men: *None*	For women: • Dinçer, Kaushal, & Grossman, 2014 (Turkey, attitudes toward gender equality) • Grépin & Bharadwaj, 2015 (Zimbabwe, attitudes toward domestic violence) • Tequame & Tirivayi, 2015 (Ethiopia, attitudes toward gender-based violence, household decision-making) For men: • Mocan & Cannonier, 2012 (Sierra Leone, attitudes toward women refusing sex with husband, disapproval of wife beating)

Employment: *Evidence for effects of increased grade attainment on employment*	For women: • Chicoine, 2012 (Kenya) • Chicoine, 2016 (Ethiopia) • Grépin & Bharadwaj, 2015 (Zimbabwe) • Keats, 2016 (Uganda)	For women: • Alam et al., 2011a, Alam et al., 2011b (Pakistan, negative effect on employment, driven by drop in unpaid labor in household) • Ali & Gurmu, 2016 (Egypt) • Baird et al., 2018 (Malawi, opportunity cost of time, share of time spent on self-employment or paid work in the last week) • Güneş, 2015 (Turkey) • Samarakoon & Parinduri, 2015 (Indonesia) • Tequame & Tirivayi, 2015 (Ethiopia) • Weitzman, 2017 (Peru) For men: • Samarakoon & Parinduri, 2015 (Indonesia, women's partners' employment)

Incarceration effect: *Evidence for effects of increased grade attainment on delayed age at first sex, first birth, or marriage (key study outcomes).*	*(See results on these outcomes in*Table 2, Table 3.1, Table 3.2, Table 4.1, Table 4.2*)*	

Media consumption: *Evidence for effects of increased grade attainment on media use/consumption.*	For women: • Agüero & Bharadwaj, 2014 (Zimbabwe, read newspapers)	For women: • Agüero & Bharadwaj, 2014 (Zimbabwe, listen to radio and watch television

Infant and child mortality: *Evidence for effects of increased grade attainment on reduced infant and child mortality. (Results reported in full in**Mensch* et al. *2018**).*	For women: • Breierova & Duflo, 2004 (Indonesia, neonatal, infant and child mortality) • Dinçer et al., 2014 (Turkey, neonatal, infant and child mortality) For men: *None*	For women: • Grépin & Bharadwaj, 2015 (Zimbabwe, infant mortality and child mortality) • Keats, 2018 (Uganda, infant mortality) • Makate & Makate, 2016 (Malawi, infant mortality and child mortality) For men: *None*

*Changing ability to achieve attitudes and preferences*		

Academic skills: *Evidence for effects of increased grade attainment on academic skills.*	For women: Behrman, 2015a, Behrman, 2015b (Malawi) • Duflo et al., 2017 (Ghana) • Keats, 2016 (Uganda) • Makate & Makate, 2016 (Malawi) • Weitzman, 2017 (Peru) For men: • Duflo et al., 2017 (Ghana)	For women: *None* For men: *None*

Knowledge of contraception: *Evidence for effects of increased grade attainment on knowledge of contraception or the fertile period.*	For women: • Agüero & Bharadwaj, 2014 (Zimbabwe, contraception) • Argaw, 2013 (Ethiopia, fertile period) • Andalón, Williams, & Grossman, 2014 (Mexico, contraception) • Dinçer et al., 2014 (Turkey, ovulation) • Keats, 2018 (Uganda, contraception)	For women: *None*

Use of contraception: *Evidence for effects of increased grade attainment on use of contraception (key study outcome).*	*(See results on these outcomes in*Table 5.1, Table 5.2*)*	

Autonomy/decision-making: *Evidence of effects of increased grade attainment on increased autonomy or decision-making within marriage or households.*	For women: • Keats, 2018 (Uganda, can refuse sex with husband, ask partner to use condom) • Samarakoon & Parinduri, 2015 (Indonesia, decision-making related to savings, children's health) • Weitzman, 2017 (Peru)	For women: • Grépin & Bharadwaj, 2015 (Zimbabwe, measure of women's empowerment) • Keats, 2018 (Uganda, other decision-making roles within marriage) • Samarakoon & Parinduri, 2015 (Indonesia, decision-making related to household expenditures, contraceptive use, children's clothing and education) • Tequame & Tirivayi, 2015 (Ethiopia, household decision-making, exception of own healthcare in urban sample)

Assortative mating: *Evidence for effects of increased grade attainment for women on spouse/partner's level of education.*	For women: • Baird et al., 2018 (Malawi, husband characteristics) • Behrman, 2015a, Behrman, 2015b (Malawi, husband years of schooling) • Behrman, 2015a, Behrman, 2015b (Malawi, spouse's level of education) • Breierova & Duflo 2004 (Indonesia, effect of difference in education on children born by age 15 and age 25) • Du, Wen & Zhao 2016 (China, spouse's level of education) • Makate & Makate, 2016 (Malawi, spouse's level of education) • Tequame & Tirivayi, 2015 (Ethiopia, age gap between respondent and spouse)	For women: • Behrman, 2015a, Behrman, 2015b (Malawi, husband age difference) • Behrman, 2015a, Behrman, 2015b (Uganda, husband age difference) • Breierova & Duflo 2004 (Indonesia, no effect of difference in education on age at marriage) • Chicoine, 2012 (Kenya, spouse's level of education) • Chicoine, 2016 (Ethiopia, husband characteristics) • Güneş, 2015 (Turkey, husband characteristics) • Güneş, 2016 (Turkey, spouse's level of education) • Tequame & Tirivayi, 2015 (Ethiopia, education gap between respondent and spouse)

Income/wealth: *Evidence for effects of increased grade attainment on women's income or household wealth.*	For women: • Baird et al., 2018 (Malawi, wage in the last three months, school girls only) • Barham et al., 2018 (Nicaragua) • Keats, 2018 (Uganda) • Du, Wen & Zhao 2016 (China) • Behrman, 2015a, Behrman, 2015b (Uganda) • Duflo et al., 2017 (Ghana) • Weitzman, 2017 (Peru)	For women: • Grépin & Bharadwaj, 2015 (Zimbabwe) • Samarakoon & Parinduri, 2015 (Indonesia) • Behrman, 2015a, Behrman, 2015b (Malawi) For men: • Duflo et al., 2017 (Ghana)

Sexual behavior: *Evidence for effect of increased grade attainment on safer sexual behavior.*	For women: • Agüero & Bharadwaj, 2014 (Zimbabwe, number of sexual partners) • Alsan & Cutler, 2013 (Uganda, virginity) • Baird et al., 2010 (Malawi, number of partners, share of partners who are older, dropouts) • Baird et al., 2012 (Malawi, had a partner over 25 years of age) • Keats, 2018 (Uganda, recent sexual activity)^a^ • Mocan & Cannonier, 2012 (Sierra Leone, HIV testing) For men: • Duflo et al., 2017 (Ghana, risky sexual behavior) • Mocan & Cannonier, 2012 (Sierra Leone, HIV testing)	For women: • Agüero & Bharadwaj, 2014 (Zimbabwe, number of sexual partners) • Baird et al., 2010 (Malawi, number of partners, frequency of sexual activity, share of partners who are older, school girls) • Baird et al., 2012 (Malawi, frequency of sexual activity, had a partner over 25 years of age) • Behrman, 2015a, Behrman, 2015b (Malawi, number of sexual partners in last 12 months) • Behrman, 2015a, Behrman, 2015b (Uganda, number of sexual partners in last 12 months) • Duflo et al., 2017 (Ghana, risky sexual behavior) • Tequame & Tirivayi, 2015 (Ethiopia, risky sexual behavior in last 12 months) For men: *None*

Other health knowledge and practices: *Evidence for effect of increased grade on improved health practices or better access to health services.*	For women: • Agüero & Bharadwaj, 2014 (Zimbabwe, knowledge of HIV/AIDS) • Baird, Garfein, McIntosh & Ozler, 2012 (Malawi, knowledge of HIV/AIDS) • Duflo et al., 2015 (Kenya, knowledge of HIV/AIDS) • Duflo et al., 2017 (Ghana, ever had unwanted pregnancy, general preventative behaviors) • Grépin & Bharadwaj, 2015 (Zimbabwe, smoking) • Güneş, 2015 (Turkey, smoking) • Samarakoon & Parinduri, 2015 (Indonesia, breastfeeding, tetanus injection, iron pills) For men: • Duflo et al., 2017 (Ghana, general preventative behaviors)	For women: • Agüero & Bharadwaj, 2014 (Zimbabwe, knowledge of HIV/AIDS) • Chicoine, 2012 (Kenya, ever terminated a pregnancy)^b^ • Keats, 2018 (Uganda, knowledge of TB, HIV/AIDS) • Makate & Makate, 2016 (Malawi, breastfeeding) For men: • Duflo et al., 2017 (Ghana, ever had/fathered unwanted pregnancy)

Note: Abdul-Salam et al. (2018), Baird et al. (2011), De Neve et al., 2015a, De Neve et al., 2015b, Ferré (2009), and Hattori et al. (2011) did not formally test pathways, but describe potential pathways in the text.

a Note that recent sexual activity, in itself, is not risky. But those engaging in recent sexual behavior may be at higher risk of negative health outcomes than those who have not engaged in recent sexual behavior if the former group adopts risky practices (e.g. no condom uses, multiple partners, etc.).

b Note that reports of having ever terminated a pregnancy may reflect more consistent use of contraception to avoid unplanned pregnancy, knowledge of and access to health services, changing attitudes about childbearing, or a combination of these factors.

Overall, Table 9 shows mixed findings for most hypothesized mechanisms, with the exception of academic skills (i.e. increased grade attainment leads to stronger academic skills), and knowledge of contraception or the fertile period, both for women.

Most of the main hypothesized mechanisms linking education and SRH in the theoretical literature are tested in one or more of these papers, but the approach to operationalizing them varies, making direct comparisons challenging. For example, identified papers use varying definitions of autonomy and decision-making, including decision-making about contraceptive use, household expenditures, children's clothing and education (Samarakoon & Parinduri, 2015), attitudes toward domestic violence (Grépin & Bharadwaj, 2015; Mocan & Cannonier, 2012; Tequame & Tirivayi, 2015), ability to refuse sex with one's partner or ask a partner to use a condom (Keats, 2016; Mocan & Cannonier, 2012), and disapproval of female genital mutilation (Mocan & Cannonier, 2012). A number of papers also test the “incarceration effect” by examining whether more education leads to delayed age at first sex, birth, or marriage (Chicoine, 2012; Tequame & Tirivayi, 2015; Verwimp, 2016), yet these definitions are not strictly consistent with the theoretical literature, which argues that these effects are independent of changing attitudes, resources, etc.

## 4 Discussion

To the best of our knowledge, this is the first systematic review summarizing evidence on the causal effects of education on sexual and reproductive health in low and middle-income countries. Many papers included in the review find evidence of a causal relationship between education and one or more outcomes, although estimated effects are often small in magnitude. However, our meta-analyses – conducted on the subset of papers with comparable exposures, outcomes and study designs – reveal more mixed results.

Overall, we find very little evidence that increased exposure to schooling leads to older age at first sex, increased use of modern contraception, or HSV-2 negative status. The results on the effects of increased schooling on age at marriage are mixed, but we find some evidence of a small effect of cash transfers conditional on school attendance on delaying marriage. Similarly, we find mixed results on the effects of education on age at first pregnancy/birth, but, similarly to marriage, we do find a small and statistically significant effect of CCTs on delaying first birth. Across all measures, we see more evidence of an effect of grade attainment on delayed birth than on delayed marriage. Consistent with these findings, we see a small but significant effect of increased exposure to schooling on lower parity/fertility. Last, while the mean effects are null, there is some indication of a negative effect of increased exposure to schooling on risk of being HIV positive.

We also find that, when authors report results from OLS models that fail to account for endogeneity in the relationship between education and SRH, as well as more rigorous models that address this source of bias, the results from the latter are attenuated substantially in nearly all cases. That is, the naïve models appear to over-state the relationship between education and sexual and reproductive health, likely due to shared drivers of both.

We propose several possible explanations for the relatively small – and often nonsignificant – mean effects of education on sexual and reproductive health. First, and most logically, the real effects of increases in grade attainment on sexual and reproductive health may be smaller than expected based on naïve associations that fail to address selectivity. This is not surprising, given the fact that improvements in both education and sexual and reproductive health at the individual and community levels likely reflect many of the same determinants, including poverty and economic stability, political will, and support for gender equality. The effects of grade attainment on SRH may also have changed over time, especially in settings where expanded access to schooling has led to the deterioration of school quality, and where many children fail to gain basic academic skills (World Bank, 2018). A clearer understanding of the mechanisms underlying the relationship between education and sexual and reproductive health – and specifically whether academic skills play an important role – would be informative in understanding the settings in which this relationship is likely to be strongest.

Second, we find considerable heterogeneity between studies included in this review, even when comparing those that measured the exposure and outcome the same way, using the same study design. Our efforts to explain the sources of heterogeneity were limited given an insufficient number of comparable studies with which to conduct moderator analyses. However, on conceptual grounds, it also makes sense that the nature of the relationship between grade attainment and sexual and reproductive health outcomes depends on numerous factors that would be reflected differently in each study, including the type of policy change or intervention implemented (e.g. were increases in school enrollment met with increased investments in school facilities?), cultural norms around adolescent marriage and childbearing, and the availability of health services in the community or country. Future research should attempt to test these important potential moderators of the relationship between education and SRH, and more research is needed using comparable study designs, settings, and outcome measures in order to draw conclusions about the conditions under which education is most likely to translate into improved sexual and reproductive health.

Third, results for many of the outcomes assessed in the included papers are likely to have been censored given varying ages at follow-up. If the effects of grade attainment on ever use of contraception, for example, are cumulative, then we may expect to see more pronounced effects for women at the end of their reproductive years, compared to younger women. Previous research has shown, for example, that women in some settings are unlikely to use contraception before their first birth, regardless of education level or access to contraception (Digitale, Psaki, Soler-Hampejsek, Barbara, & Mensch, 2017). Therefore, a positive effect of education on contraceptive use may not emerge until women have reached their desired fertility. Similarly, in each paper assessing the effects of education on HIV status, HIV negative women were still at risk at follow-up; that is, the difference by education level may diminish by older ages. While we attempted to account for age at follow-up in our moderator analyses, few of the studies included reported separate results by age group.

Last, previous research has highlighted the challenges in accurately measuring the timing of life events, especially those that are self-reported (Mensch, Soler-Hampejsek, Kelly, Hewett, & Grant, 2014). We might expect less error in reporting total fertility and HIV status (from testing data), than in age at first sex, for example. However, important areas of potential bias exist for those outcomes as well, such as underreporting of children who have died, or refusal of HIV testing among those who are at highest risk of STIs. However, our findings on a small but significant mean effect of increased grade attainment on lower fertility may reflect the cumulative effects of changes in other outcomes, such as age at marriage and first birth, which are on the pathway between grade attainment and fertility.

### 4.1 Opportunities for future research

This review identified two important gaps in the existing evidence on the relationships of interest: 1) the narrow focus on the effects of grade attainment on health, rather than other aspects of schooling, in particular, literacy or numeracy and school quality; and 2) insufficient focus on the effects of men's education on health outcomes.

Identified papers were limited in their definition of education. Each of the 35 papers included in this review examined the effects of access to school (grade attainment, attendance, number of years of school attended) on health outcomes. We were unable to identify any papers investigating the effects of age at school leaving, learning outcomes, socialization in the school environment, or other aspects of the formal education experience. This pattern is likely due, in part, to the fact that most of the papers identified take advantage of natural experiments, and many use Demographic and Health Survey data (Corsi, Neuman, Finlay, & Subramanian, 2012), which lack information on many other aspects of education. The most commonly used natural experiments, such as policy changes that eliminated school fees, may have the clearest direct effects on grade attainment. However, it is possible that those policy changes also significantly increased learning via increased grade attainment. While several papers investigated the effect of increased schooling on literacy, we are unable to tease out the independent effects of grade attainment and improvements in learning on health outcomes. Further, although several papers did attempt to identify threshold effects of grade attainment (e.g. 8 or more years of schooling), we did not identify enough papers that categorized grade attainment this way to draw any conclusions.

Some of the identified papers examined the effects of exposure to policies or interventions aimed at improving school quality, in part. Even in those cases, however, the authors estimated the causal relationship between grade attainment and SRH outcomes, rather than the effects of school quality on SRH outcomes. The focus on the effects of access to school, rather than other indicators of education, is an important gap in the literature, particularly in an environment of inadequate or declining school quality. This gap could potentially be filled through analyses of existing data, or investments in the collection and analysis of new data on existing programs.

Second, our review only identified four papers examining the effects of men's education on sexual and reproductive health. The research focus on delaying events in women's lives (e.g. age at first birth, age of marriage) may reflect the fact that these events tend to occur at younger ages for women, when risks to health and wellbeing may be greater. The few papers that did include estimates for men (all of which included women as well) were much less likely to find significant effects in the expected direction for men than for women. However, more evidence is needed to draw conclusions about these relationships.

These important gaps in evidence underline the fact that the lack of significant effects in some papers does not, in itself, imply that these relationships do not operate in the expected direction. For example, it is possible that learning has a strong positive effect on health outcomes, and that men's education has a significant beneficial effect on sexual and reproductive health outcomes. This review simply did not identify evidence demonstrating these relationships. Similarly, there may be positive ripple effects of education on other members of the household or community (De Neve & Kawachi, 2017), but our review did not identify any papers examining these relationships.

Although our review was not designed to systematically evaluate the evidence on mechanisms linking education and sexual and reproductive health, many authors of included studies tested mechanisms empirically, and we have summarized their findings. Studies investigating correlations consistently show that more education is associated with each of these outcomes. In fact, since many of the papers included in our review report significant results on the causal relationship between grade attainment and at least one SRH outcome, we might expect analyses on mechanisms from these papers to be biased toward finding significant effects. It is somewhat striking, therefore, that the evidence is so mixed for relationships such as the effect of grade attainment on fertility preferences, autonomy and decision-making, and sexual and other health behaviors. We note, however, that despite agreement on hypothesized mechanisms, authors of included studies operationalize those mechanisms in different ways, and even those papers investigating the same relationship between education and SRH did not always test the same hypothesized mechanisms. Further, as previously noted, the fact that education has a significant effect on the intervening variables, e.g. child mortality, does not necessarily mean that this is a pathway – or certainly the *main* pathway – through which education affects fertility or other outcomes. A more appropriate approach might be to test the effects of education on age at each event, while attempting to control for the other likely pathways.

### 4.2 Strengths and limitations

This review has numerous strengths. Many researchers have demonstrated significant associations between education and health outcomes. However, the inclusion criteria for this review were chosen to identify a pool of papers that came as close as possible to estimating the true causal relationships. We believe, therefore, that our results provide more valid estimates of the relationships between education and sexual and reproductive health outcomes than previous work focused only on associations. The inclusion of working papers from several reputable sources allowed us to incorporate potentially newer relevant findings from studies that have not yet been published in scientific journals. Last, in addition to conducting a systematic review of the literature on our research question, we ran meta-regressions when sufficient data were available to estimate mean effects by outcome, as well as moderator analyses to attempt to identify sources of heterogeneity between studies.

This review also has several key limitations. In order to ensure that the scope was manageable, we selected a core set of key outcomes related to sexual and reproductive health. As a result, some potentially relevant pathways and outcomes were excluded, such as experience of sexual violence, or termination of a pregnancy. We set strict inclusion criteria to ensure that identified studies provide evidence of causal relationships between education and SRH outcomes. Some excluded studies with relatively weaker designs or analytical approaches may nonetheless provide useful information on relationships of interest. Though we were able to calculate mean effect sizes for a subset of outcomes, the small number of comparable studies per outcome meant that we could not determine what characteristics may be driving these overall effects (e.g. potential study- and macro-level moderators such as minimum age at the time of survey or primary completion rate) (Hempel et al., 2013). This review was designed to focus on the relationships between education and a set of key outcomes, and a systematic assessment of the mechanisms underlying these relationships was beyond the scope. Although the authors of included papers provide some theoretical and empirical insights into the likely mechanisms at play, a more systematic review of the mechanisms underlying each relationship is warranted.

## 5 Conclusions

This systematic review on the effects of education on sexual and reproductive health provides evidence of the benefits of policies designed to eliminate primary school fees, not only in terms of increasing school enrollment and grade attainment, but also for improving SRH outcomes in certain circumstances, especially fertility and HIV status. Many of the policy changes exploited as natural experiments focused on elimination of school fees at the primary level. Although elimination of secondary school fees is more recent, and not yet as widespread (World Bank, 2018), there is reason to believe, based on our findings, that those policies will also have substantial positive effects on education outcomes, and possibly health outcomes, particularly if sufficient investments are made in maintaining or improving the quality of schooling.

Our review provides evidence of the potential for positive ripple effects of investments in increased schooling, with the caveat that those investments may not translate into improved health as consistently as expected by many policy-makers. Further, while national policy changes are one important approach, there may also be more regionally or community-focused programs, or policies aimed at increasing enrollment for specific groups, with the potential to have these ripple effects. And yet for some groups, particularly those least likely to attend school, policy changes alone may not be sufficient to ensure universal school enrollment and completion of secondary school. More intense efforts may be needed for those groups, and the benefits may be different (either smaller or larger) than the benefits for those who attend school solely as a result of a policy change eliminating school fees.

An accurate understanding of the extent to which improvements in education are likely to lead to better health outcomes is essential for achievement of global development goals. Sustainable Development Goal 4 aims to ensure inclusive and quality education for all, and to promote lifelong learning (United Nations, 2015). Goal 3 focuses on improving health and well-being, including reducing neonatal, child and maternal mortality, and integrating reproductive health into national strategies. International policy frameworks recognize the potential synergy between these goals (UNESCO, 2016). And yet, although the narrative in the development field has shifted in recent years away from single sector interventions toward “multi-sectoral” or “integrated” policies and programs, in practice funding streams and policy-making decisions are still often siloed – especially with regard to education and health. This review underlines the fact that governments, practitioners and donors interested in improving health outcomes should integrate investments in education into their portfolios but should not assume that increasing access to school alone will be sufficient to achieve improvements in health. Further, those working in education should seek to systematically document the benefits of their policies and programs, in terms of both education and health.

## Ethics approval statement

This manuscript reports the results of a systematic review of the existing literature on the effects of education on sexual and reproductive health in low and middle-income countries. No new data collection with human subjects was done as part of this work. The full study protocol is available online (PROSPERO registry # CRD42017073224).

## Conflicts of interest

The authors confirm that have no conflicts of interest to disclose.

## Footnotes

1 Although most of the literature refers to exposure to formal schooling explicitly or implicitly, it also includes learning (e.g. literacy or numeracy), which may result from formal (in the school system), nonformal (organized programs outside of the school system), and/or informal (in the course of daily life) education. For the purposes of this review, we refer to both exposure to formal schooling, and learning that may result from different types of education.

2 The full list of search terms is included in the study protocol, published online (PROSPERO registry# CRD42017073224).

4 The full study protocol is published online (PROSPERO registry# CRD42017073224).

5 Most authors used an alpha level of 0.10 in their assessments of statistical significance, although we report partial correlations and 95% confidence intervals in forest plots. As a result, in rare cases the authors' assessments of significance and our assessments based on partial correlation coefficients may conflict.

6 There were two studies (Alam, Baez, & Del Carpio, 2011; Alsan & Cutler, 2013) where the authors could not be reached, and we did not have sufficient information to convert those effect sizes.

7 Throughout the text and tables we refer to the comparison between OLS models and “more rigorous models”. We use the latter term to refer to models that adopt analytical approaches designed to address the endogeneity in the relationship between education and sexual and reproductive health (e.g. RCTs or quasi-experimental studies using a regression discontinuity design), as laid out in our inclusion criteria in our study protocol.

8 The results on the effects of education on maternal and child health are reported elsewhere (Mensch et al., 2018).

9 Note that, for all meta-regressions that include the results from Baird et al., 2018, we present results in the main tables that include both school girls and dropouts subgroups because the differential results were key to the overall findings. Where possible, we defer to Baird et al., 2018 because this paper evaluated the long-term effects of the intervention. We also conduct robustness checks utilizing the results from the other Baird et al. papers, which can be found in Supplementary Table S9.

10 Note that technically measurements of total pregnancies (rather than total pregnancies reaching viable gestational age) would be considered gravidity, not parity; however, for simplicity we use the term parity/fertility.

11 This result is due to the large heterogeneity between studies, which increases uncertainty regarding the mean of the distribution of population effects.

Appendix A Supplementary data to this article can be found online at https://doi.org/10.1016/j.ssmph.2019.100386.

## Footnotes

3 The types of natural experiments and other quasi-experimental studies that were ultimately included were: two-stage least squares, IV poisson, IV probit, regression discontinuity (both fuzzy and sharp) and differences-in-differences. We did not identify studies that controlled for endogeneity for inclusion outside of these methods in our search.
