# Supplementary material for: Causal effects of education on sexual and reproductive health in low and middle-income countries: A systematic review and meta-analysis
Source: SSM Popul Health. 2019 May 20;8:100386. doi: 10.1016/j.ssmph.2019.100386 (PMC6582211; doi:10.1016/j.ssmph.2019.100386)
Supplement: Multimedia component 2 [file mmc2.docx]

***Supplementary Information***

**Table S1.** Risk of bias assessment results by study

|  |  |  | **RCTs** | **Quasi-Experimental** | | | |  |  |  |  |  |
| --- | --- | --- | --- | --- | --- | --- | --- | --- | --- | --- | --- | --- |
| **Author(s)/year** | **Study Type** | **Selection Bias** | **Inclusion/**  **Exclusion Criteria** | **Regression Discontinuity** | **Natural Experiments** | **Heckman and IV** | **Panel Data** | **Sample Size** | **Confounding** | **Attrition** | **Mechanisms** | **Score** |
| Alam, Baez and Del Carpio (2011) | Quasi-experimental | 1 |  | 1 |  |  | 1 | 0 | 0 | 0 | 1 | 3 |
| Ali & Gurmu (2016) | Quasi-experimental | 1 |  | 1 | 1 |  |  | 0 | 0 | 0 | 1 | 3 |
| Andalón, Williams, & Grossman (2014) | Quasi-experimental | 1 |  |  | 1 | 1 |  | 0 | 1 | 0 | 1 | 4 |
| Argaw (2013) | Quasi-experimental | 1 |  |  | 0 | 0 | 1 | 0 | 1 | 0 | 1 | 3 |
| Baird, Garfein, McIntosh, & Ozler (2012) | Experimental | 1 | 1 |  |  |  |  | 1 | 1 | 1 | 1 | 6 |
| Baird, McIntosh, & Ozler (2010) | Experimental | 1 | 1 |  |  |  |  | 1 | 1 | 1 | 1 | 6 |
| Baird, Chirwa, McIntosh, & Ozler (2010) | Experimental | 1 | 1 |  |  |  |  | 0 | 0 | 1 | 1 | 4 |
| Behrman (2015) | Quasi-experimental | 1 |  | 1 | 1 | 0 |  | 0 | 1 | 0 | 1 | 3 |
| Breierova & Duflo (2003) | Quasi-experimental | 1 |  |  | 1 | 0 | 1 | 0 | 0 | 0 | 1 | 2 |
| Chicoine (2012) | Quasi-experimental | 1 |  |  | 1 | 1 |  | 0 | 0 | 0 | 1 | 3 |
| De Neve, Fink, Subramanian, & Moyo (2015) | Quasi-experimental | 1 |  |  | 1 | 0 |  | 0 | 1 | 0 | 1 | 3 |
| Dinçer, Kaushal, Grossman (2013) | Quasi-experimental | 1 |  |  | 1 | 1 |  | 0 | 1 | 0 | 1 | 4 |
| Du, Wen, & Zhao (2016) | Quasi-experimental | 1 |  |  | 1 | 1 |  | 0 | 0 | 0 | 1 | 3 |

| Duflo, Dupas, & Kremer (2015) | Experimental | 1 | 1 |  |  |  |  | 0 | 1 | 1 | 1 | 5 |
| --- | --- | --- | --- | --- | --- | --- | --- | --- | --- | --- | --- | --- |
| Duflo, Dupas, & Kremer (2017) | Experimental | 1 | 1 |  |  |  |  | 0 | 1 | 1 | 1 | 5 |
| Grant (2015) | Quasi-experimental | 1 |  |  | 0 | 0 |  | 0 | 0 | 0 | 1 | 2 |
| Grepin & Bharadwaj (2015) | Quasi-experimental | 1 |  | 1 | 1 | 0 |  | 0 | 0 | 0 | 1 | 2 |
| Gunes (2015) | Quasi-experimental | 1 |  |  | 1 | 1 |  | 0 | 0 | 0 | 1 | 3 |
| Gunes (2016) | Quasi-experimental | 1 |  |  | 1 | 1 |  | 0 | 0 | 0 | 1 | 3 |
| Hattori (2011) | Quasi-experimental | 1 |  |  | 0 | 0 |  | 0 | 1 | 0 | 1 | 3 |
| Keats (2016) | Quasi-experimental | 1 |  | 1 | 0 | 0 |  | 0 | 0 | 0 | 1 | 2 |
| Makate & Makate (2016) | Quasi-experimental | 1 |  | 1 | 1 | 0 |  | 0 | 1 | 0 | 1 | 3 |
| Mocan & Cannonier (2012) | Quasi-experimental | 1 |  |  | 0 | 1 |  | 0 | 1 | 0 | 1 | 3 |
| Osili & Long (2008) | Quasi-experimental | 1 |  |  | 1 | 0 |  | 0 | 0 | 0 | 0 | 1 |
| Samarakoon & Parinduri (2015) | Quasi-experimental | 1 |  | 1 | 1 |  |  | 0 | 1 | 0 | 1 | 4 |
| Tequame & Tirivayi (2014) | Quasi-experimental | 1 |  | 1 | 1 | 0 |  | 0 | 1 | 0 | 1 | 3 |
| Verwimp (2016) | Quasi-experimental | 1 |  | 1 |  | 0 |  | 1 | 0 | 0 | 1 | 3 |
| Weitzman (2017) | Quasi-experimental | 1 |  | 1 | 1 |  |  | 0 | 0 | 0 | 1 | 3 |

**Table S2.** Results for boys and men

| **Authors (Year)** | | **Country** | **Age Group** | **Education**  **Exposure** | **Expected/ Unexpected/Null: OLS models** | **Expected/ Unexpected/Null: More rigorous models** | **Reported effects: OLS models**  **(95% CI)** | **Reported effects: More rigorous models  (95% CI)** |
| --- | --- | --- | --- | --- | --- | --- | --- | --- |
| **Ever married/cohabited (dichotomous)** | | | | | | | | |
|  | Duflo, Dupas & Kremer (2015)ˠ‡ | Kenya | Primary school-age (avg 13) at baseline | CCT on primary schooling (dichotomous) | Not calculated | Null | Not provided | -0.008  (-0.016, 0.000) |
|  | Duflo, Dupas & Kremer (2017)ˠ | Ghana | Secondary school-age (avg 17) at baseline | Years of schooling (continuous) | Not calculated | Null | Not provided | -0.033  (-0.078, 0.012) |
| **Ever pregnant/gave birth (dichotomous)** | | | | | | | | |
|  | Duflo, Dupas & Kremer (2015)ˠ‡ | Kenya | Primary school-age (avg 13) at baseline | CCT on primary schooling (dichotomous) | Not calculated | Null | Not provided | -0.002  (-0.008, 0.003) |
|  | Duflo, Dupas & Kremer (2017)ˠ | Ghana | Secondary school-age (avg 17) at baseline | Years of schooling (continuous) | Not calculated | Null | Not provided | -0.020  (-0.075, 0.035) |
| **First birth by age 16 (dichotomous)** | | | | | | | | |
|  | Duflo, Dupas & Kremer (2015)ˠ‡ | Kenya | Primary school-age (avg 13) at baseline | CCT on primary schooling (dichotomous) | Not calculated | Null | Not provided | -0.005  (-0.013, 0.003) |
| **Total births/children (continuous)** | | | | | | | | |
|  | Duflo, Dupas & Kremer (2017)ˠ | Ghana | Secondary school-age (avg 17) at baseline | Years of schooling (continuous) | Expected | Null | -0.074  (-0.101, -0.047) | -0.025  (-0.103, 0.053) |
| **Currently using contraception (dichotomous)** | | | | | | | | |
|  | Duflo, Dupas & Kremer (2015)ˠ‡ | Kenya | Primary school-age (avg 13) at baseline | CCT on primary schooling (dichotomous) | Not calculated | Null | Not provided | -0.041  (-0.084, 0.002) |
| **Ever used contraceptives (dichotomous)** | | | | | | | | |
|  | Mocan & Cannonier (2012) | Sierra Leone | 15-18, 22-28 | Grade attainment (continuous) | Not calculated | Expected | Not provided | 0.069  (-0.120, -0.018) |
| **HIV positive status (dichotomous)** | | | | | | | | |
|  | De Neve, Fink, Subramanian, Moyo, & Bor (2015) | Botswana | 15-49 | Years of schooling (continuous) | Not calculated | Null | Not provided | -0.05  (-0.107, 0.007) |
|  | Duflo, Dupas & Kremer (2015)ˠ‡ | Kenya | Primary school-age (avg 13) at baseline | CCT on primary schooling (dichotomous) | Not calculated | Null | Not provided | 0.001  (-0.005, 0.003) |
| **HSV-2 positive status (dichotomous)** | | | | | | | | |
|  | Duflo, Dupas & Kremer (2015)ˠ‡ | Kenya | Primary school-age (avg 13) at baseline | CCT on primary schooling (dichotomous) | Not calculated | Null | Not provided | 0.005  (-0.023, 0.013) |

ˠ Studies that received a risk of bias score of 4 or higher.

† Results were converted to Partial correlations using more direct estimates.

‡ Studies that did not clear the direct causal pathway, but are otherwise rigorous (e.g. RCTs that run OLS models).

Note: Included in forest plots are studies where the exposure and outcomes are comparable. Age groups are from the time of the survey unless otherwise specified.

**Appendix S3.** Model types and formulas used for conversion to Partial correlations

Note:

- IV here refers to independent variable, NOT instrumental variable
- DV refers to the dependent variable
- For all two-stage regressions, the second stage regressor is referenced as the IV
- $r$ refers to the partial correlation
- Equation 1.1 was applied to all results with continuous DVs to improve comparability, unless the equation didn’t properly fit the reported models. In those cases, we applied Equations 1.2.1-2.3 based on the bolded criteria below.

**Continuous DV**

- **Linear models with either Continuous or Dichotomous IVs**
  - Equation 1.1:
    - Equations:
      - $t=\frac{B}{{se}_{B}}$, where $t$ refers to the t-statistic
      - $r=\frac{t}{\sqrt{t^{2}+df}}$
    - Data needed:
      - T-statistic ($t$)or Unstandardized Regression Coefficient and Standard Error ($B, {se}_{B}$)
      - Residual Degrees of Freedom (sample size minus the number of predictors) ($df$)

**Dichotomous DV**

- **Logit Models**
- Equation 2.1: Logit models with dichotomous IV and dichotomous DV
  - Equations:
    - $B=log(OR)$
    - $d=B(\frac{\sqrt{3}}{\pi})$ , where $d$ refers to Cohen’s d
    - $r=\frac{d}{\sqrt{4+d^{2}}}$
  - Data needed:
    - Unstandardized Regression Coefficient or Odds Ratio ($B$ or $OR$)
- **Linear models with dichotomous IVs**
- Equation 2.2.1: Linear models with dichotomous IV and dichotomous DV (if control group success proportion is presented)
  - Equations:
    - $a=n_{treat}(p_{control}+B)$
    - $b=n_{treat}(1-(p_{control}+B))$
    - $c=n_{control}*p_{control}$
    - $d=n_{control}(1-p_{control})$
    - $r=\frac{\left( ad \right)-(bc)}{\sqrt{(a+b)(c+d)(a+c)(b+d)}}$
  - Data needed:
    - Unstandardized Regression Coefficient ($B$)
    - Control group sample size ($n_{treat}$)
    - Treatment group sample size ($n_{control}$)
    - Control group success proportion (i.e. mean) of DV ($p_{control}$)
- Equation 2.2.2: Linear models with dichotomous IV and dichotomous DV (if only overall success proportion is presented)
  - Equations:
    - $a=n_{treat}(p+.5B)$
    - $b=n_{treat}(1-(p+.5B))$
    - $c=n_{control}(p-.5B)$
    - $d=n_{control}(1-(p-.5B))$
    - $r=\frac{\left( ad \right)-(bc)}{\sqrt{(a+b)(c+d)(a+c)(b+d)}}$
  - Data needed:
    - Unstandardized Regression Coefficient ($B$)
    - Control group sample size ($n_{control}$)
    - Treatment group sample size ($n_{treat}$)
    - Overall success proportion (i.e. mean) of DV ($p$)
- **Probit Models**
- Imputed 0 if regression coefficient=0, otherwise:
- Equation 2.3: Probit models
  - Equation:
    - $d=\frac{B}{{SD}_{x}}$
    - $r=\frac{d}{\sqrt{r+d^{2}}}$
  - Data needed:
    - Unstandardized Regression Coefficient ($B$)
    - Standard Deviation of IV (either for the entire analytical sample or disaggregated by treatment and control groups) (${SD}_{x}$)

**Standard errors and Confidence Intervals**

- **Standard errors**
  - If only the standard error of the coefficient is available:
    - Equation 3.1:
      - ${se}_{r}=\frac{r*{se}_{B}}{B}$, where ${se}_{r}$ refers to the standard error of the Partial correlation
    - Data needed
      - Unstandardized Regression Coefficient ($B$)
      - Standard Error of the Unstandardized Regression Coefficient (${se}_{B}$)
  - If only the 95% confidence intervals for the coefficient are available:
    - Equation 3.2:
      - ${se}_{B}=\frac{{CI}_{upper}-{CI}_{lower}}{1.96}$, where ${se}_{B}$ refers to the standard error of the unstandardized regression coefficient
      - ${se}_{r}=\frac{r*{se}_{B}}{B}$, where ${se}_{r}$ refers to the standard error of the Partial correlation
    - Data needed
      - Unstandardized Regression Coefficient ($B$)
    - Confidence intervals of the Unstandardized Regression Coefficient (${CI}_{upper}$, ${CI}_{lower}$)
- **Confidence intervals**
  - The equation below can apply to either regression coefficients as well as partial correlations:
    - Equation 3.3
      - $CI=B\pm{se}_{B}\cdot1.96$
